# AI-driven computational methods and benchmarking for T-cell antigen identification

**DOI:** 10.1093/bib/bbag123

**Published:** 2026-03-17

**Authors:** Yang Deng, Jinhao Que, Guangfu Xue, Yideng Cai, Wenyi Yang, Yilin Wang, Yi Hui, Zuxiang Wang, Yi Lin, Wenyang Zhou, Zhaochun Xu, Qinghua Jiang, Haoxiu Sun

**Affiliations:** Center for Bioinformatics, School of Life Science and Technology, Harbin Institute of Technology, 92 Xidazhi Street, Nangang District, Harbin, 150000 Heilongjiang Province, China; Center for Bioinformatics, School of Life Science and Technology, Harbin Institute of Technology, 92 Xidazhi Street, Nangang District, Harbin, 150000 Heilongjiang Province, China; Center for Bioinformatics, School of Life Science and Technology, Harbin Institute of Technology, 92 Xidazhi Street, Nangang District, Harbin, 150000 Heilongjiang Province, China; Center for Bioinformatics, School of Life Science and Technology, Harbin Institute of Technology, 92 Xidazhi Street, Nangang District, Harbin, 150000 Heilongjiang Province, China; Center for Bioinformatics, School of Life Science and Technology, Harbin Institute of Technology, 92 Xidazhi Street, Nangang District, Harbin, 150000 Heilongjiang Province, China; Center for Bioinformatics, School of Life Science and Technology, Harbin Institute of Technology, 92 Xidazhi Street, Nangang District, Harbin, 150000 Heilongjiang Province, China; Center for Bioinformatics, School of Life Science and Technology, Harbin Institute of Technology, 92 Xidazhi Street, Nangang District, Harbin, 150000 Heilongjiang Province, China; School of Interdisciplinary Medicine and Engineering, Harbin Medical University, 157 Baojian Road, Nangang District, Harbin, 150076 Heilongjiang Province, China; School of Interdisciplinary Medicine and Engineering, Harbin Medical University, 157 Baojian Road, Nangang District, Harbin, 150076 Heilongjiang Province, China; School of Interdisciplinary Medicine and Engineering, Harbin Medical University, 157 Baojian Road, Nangang District, Harbin, 150076 Heilongjiang Province, China; School of Interdisciplinary Medicine and Engineering, Harbin Medical University, 157 Baojian Road, Nangang District, Harbin, 150076 Heilongjiang Province, China; Center for Bioinformatics, School of Life Science and Technology, Harbin Institute of Technology, 92 Xidazhi Street, Nangang District, Harbin, 150000 Heilongjiang Province, China; School of Interdisciplinary Medicine and Engineering, Harbin Medical University, 157 Baojian Road, Nangang District, Harbin, 150076 Heilongjiang Province, China; School of Interdisciplinary Medicine and Engineering, Harbin Medical University, 157 Baojian Road, Nangang District, Harbin, 150076 Heilongjiang Province, China

**Keywords:** mRNA vaccines, T-cell antigen identification, artificial intelligence, benchmarking

## Abstract

The rise of mRNA vaccines highlights the pivotal role of T-cell antigen identification in modern vaccinology and personalized medicine. T-cell recognition relies on the sophisticated ternary interaction between the T-cell receptor (TCR), the major histocompatibility complex (MHC) molecule, and the peptide antigen, which forms the peptide–MHC (pMHC) complex. Computational methods, particularly artificial intelligence (AI), are indispensable for accurately predicting these complex bindings. This review systematically surveys the rapidly evolving AI-driven landscape for T-cell antigen identification, providing a comprehensive categorization of methods for MHC-I, MHC-II, and the highly complex TCR–pMHC binding prediction, alongside foundational data resources. Crucially, we conduct a rigorous, standardized benchmarking of 18 state-of-the-art TCR–pMHC prediction models across diverse training data sources. Our evaluation on two distinct and challenging out-of-distribution (OOD) unseen epitope variant datasets reveals a significant and concerning generalization gap in current predictors. Notably, the overall absolute predictive gain remains marginal across all models under OOD conditions. This result underscores a severe and persistent generalization challenge when faced with novel epitope variants. To address these limitations, we emphasize the urgent need for enhanced structural modeling, the integration of multi-omics data, and the development of generative models for *de novo* TCR design. By advancing these computational frontiers, our community can accelerate the transition from prediction to rational design in immunoinformatics.

## Introduction

The remarkable success of mRNA vaccines [1], especially their key role in fighting the COVID-19 pandemic [2, 3], highlights their transformative potential in modern vaccinology and medicine [4–6]. Beyond infectious diseases, mRNA vaccine development is also demonstrating broad prospects for personalized treatment options for various malignant tumors, with promising clinical progress observed in areas such as melanoma [7], glioblastoma [8], colorectal cancer [9, 10], and pancreatic cancer [11, 12]. These vaccines use the host’s cellular mechanisms to produce specific antigens, thereby triggering a strong immune response [13, 14]. The core of this protective immunity is the activation of T cells, which play a crucial role in the elimination of infected or abnormal cells and the long-term immune memory [15, 16]. T cells recognize antigens only when presented by molecules from the major histocompatibility complex (MHC, known as human leukocyte antigen (HLA) in humans) on the cell surface, a process mediated by their highly specific T-cell receptor (TCR) [17, 18]. Accurately identifying these T cell antigens (and distinguishing tumor-reactive T cells from bystanders [19]) is crucial for designing effective mRNA vaccines and other immune therapies [20, 21], but traditional experimental methods often require a significant amount of manpower and resources, and have long cycles.

**Figure 1:**
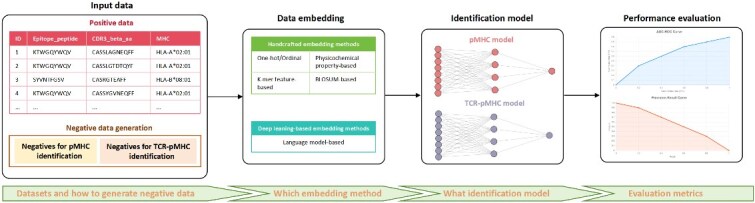
The conceptual framework for AI-driven T-cell antigen identification. This diagram illustrates the systematic process of identifying T-cell antigens using AI. It outlines the flow from Input data (positive and generated negative samples), through Data embedding (various sequence representation methods), to the Identification model (for pMHC and TCR–pMHC binding predictions), and finally to Performance evaluation using relevant metrics. This framework highlights the key computational stages raised in this review.

Driven by advances in high-throughput TCR sequencing [22–24], mass cytometry [25–27], microfluidics [28–30] as well as the accumulation of antigen peptide–MHC (pMHC)–TCR binding data [31–33], computational approaches, especially artificial intelligence (AI) methods, have emerged as indispensable tools for accelerating the discovery of T cell antigens [34–36]. Although several existing reviews have also summarized some computational methods for T-cell antigen identification, such as [34–39], they often lack comprehensive coverage, omitting the most recent advancements [34, 35, 37] or not exclusively focusing on T-cell antigen recognition [36, 37], and not specifically summarizing those methods of binding of MHC class II molecules to antigenic peptides [35, 36, 39]. In addition, some of those reviews are not constructed from a technical perspective [34], which constitutes an entry barrier for AI researchers seeking in-depth research in this field. It is worth noting that a recent comprehensive review [40] also provides a comprehensive summary of computational methods and datasets for predicting neoantigens from a computational perspective. However, this previous work does not wholly present the entire T-cell antigen recognition process, notably lacking a complete introduction to critical steps such as data embedding methods and negative sample generation strategies. In contrast, the distinct value of this review lies in its **dual contribution**. First, it adopts an AI researcher’s perspective, systematically investigating the field through a coherent framework that includes task definitions, available datasets, AI models, evaluation indicators, and forward-looking prospects. Second, moving beyond a traditional survey, this work presents a rigorous, standardized benchmarking of 18 state-of-the-art (SOTA) TCR–pMHC prediction models. This empirical evaluation, particularly on challenging out-of-distribution (OOD) variant datasets, allows us to precisely identify the critical “generalization gap” and provide data-driven insights for future development. Recent comprehensive assessments [41] have begun to reveal the generalization constraints of current TCR–epitope prediction methods. Building upon this emerging consensus, our review utilizes a rigorous OOD benchmarking not merely to rank models, but to diagnose the fundamental limitations of sequence-based paradigms. We demonstrate that the persistent generalization gap—confirmed by our stress tests on unseen variants—signals a critical inflection point for the field: the necessity to move beyond black-box prediction toward structure-informed modeling and generative design. This review is expected to provide valuable guidance for AI researchers, with the aim of entering this key field quickly and effectively. The general conceptual framework for this review is illustrated in Fig. 1.

## T-cell antigen recognition: a multi-step process

The recognition of T-cell antigens is a pivotal multi-step process fundamental to adaptive immunity, serving as a critical upstream challenge for AI in designing effective immunotherapies. This intricate pathway begins with antigen-presenting cells (APCs), such as dendritic cells, macrophages, or B cells, which play a crucial role in capturing and processing antigens. Within APCs, antigens are degraded into smaller peptide fragments. Subsequently, these peptides must bind to MHC molecules, which then present the peptide on the cell surface. This antigen presentation mechanism enables the adaptive immune system to meticulously survey the host cell’s protein landscape for signs of pathogens or mutations.

These MHC molecules are broadly categorized into two predominant classes [42]: MHC class I and MHC class II. MHC class I molecules are expressed on nearly all nucleated cells and primarily present endogenous antigen peptides derived from proteins synthesized inside the cell, such as viral or tumor proteins [43]. Structurally, MHC I molecules are heterodimers composed of a heavy chain (encoded by classical HLA-A, HLA-B, or HLA-C genes in humans), noncovalently associated with $\beta $2-microglobulin (B2M) [44]. This structure forms a peptide-binding groove that typically accommodates short peptides, ranging from 8 to 11 amino acids. This characteristic defines the input peptide length crucial for MHC I-peptide binding prediction models. By contrast, MHC class II molecules are predominantly found on professional APCs, such as dendritic cells, macrophages, and B cells [43]. They present exogenous antigen peptides derived from proteins captured from outside the cell, such as bacterial components. Structurally, MHC II molecules are also heterodimers, composed of two membrane-anchored chains, an $\alpha $-subunit and a $\beta $-subunit (encoded by classical HLA-DP, HLA-DQ, and HLA-DR genes in humans) [45]. Their peptide-binding groove is more open-ended, allowing them to accommodate larger peptides [46], typically around 10–16 residues in length, though shorter or longer lengths are not uncommon [45, 47]. The distinct structural features and peptide length preferences of MHC classes I and II molecules necessitate specialized computational approaches for accurate binding prediction [48, 49].

**Figure 2:**
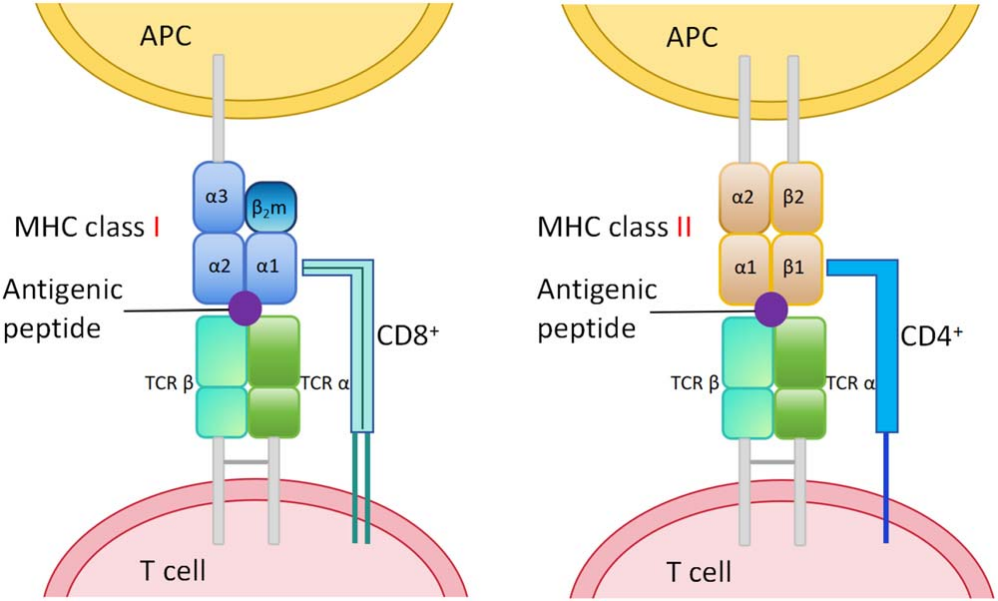
Schematic diagram of antigen presentation and recognition. This figure illustrates how T cells recognize antigens presented by MHC class I (left) and MHC class II (right) molecules on APCs. MHC class I, bound to a peptide antigen, interacts with a TCR and the CD8$^{+}$ co-receptor. MHC class II, also presenting an antigenic peptide, engages with a TCR and the CD4$^{+}$ co-receptor.

The precise identification of T-cell antigens hinges upon two sequential and highly specific molecular recognition events [43, 50]. The first step involves the formation of the pMHC complex. Following antigen processing, the resulting peptide fragments must bind to the MHC molecules. This binding is highly selective, dependent on the peptide’s sequence and length, as well as the specific MHC allele expressed by the cell. These newly formed pMHC complexes are then presented on the cell surface. Specifically, pMHC I complexes are primarily recognized by CD8$^{+}$ cytotoxic T cells [51], while pMHC II complexes are predominantly recognized by CD4$^{+}$ helper T cells [52]. The immense diversity of MHC alleles and potential peptide sequences makes experimental determination of all possible pMHC binding events infeasible. Accurate prediction of peptide–MHC binding affinity and stability is therefore the critical initial computational challenge in identifying potential T-cell antigens. The second step of T-cell antigen recognition centers on the TCR. T cells, through their unique TCRs, specifically recognize and bind to these pMHC complexes displayed on the APC surface. This TCR–pMHC interaction forms a highly intricate interface, where the TCR simultaneously engages both the peptide antigen and specific regions of the MHC molecule. The strength and duration of this precise interaction dictate the subsequent activation of the T cell and the initiation of a targeted immune response. Therefore, accurately predicting TCR–pMHC binding, and ultimately the immunogenicity of a given antigen, represents the second major computational challenge in the comprehensive identification of T-cell antigens. The schematic diagram of the entire recognition process is shown in Fig. 2.

## Publicly available datasets for T-cell antigen recognition

The rapid advancement of AI methods in T-cell antigen identification heavily relies on the availability of high-quality, publicly accessible datasets, which serve as the foundational training and validation resources for predictive models. As discussed in Section “T-cell antigen recognition: a multi-step process,” T-cell antigen recognition fundamentally comprises two sequential processes: the binding of antigenic peptides to MHC molecules, followed by the recognition of these pMHC complexes by TCRs. Accordingly, this section will separately present the common publicly available datasets relevant to peptide–MHC binding (encompassing both MHC class I and MHC class II interactions) and those of TCR–pMHC binding. Besides, we will also introduce the common strategies used for generating negative samples.

### Datasets for peptide–MHC class I binding

The development of robust AI models for peptide–MHC class I binding prediction has benefited significantly from a growing collection of experimentally determined binding data. The Immune Epitope Database (IEDB) [33] stands as the most comprehensive public repository, archiving thousands of experimentally determined binding affinities for various peptides to diverse MHC class I alleles across multiple species, including humans (HLA-A, -B, -C). These data are typically generated through *in vitro* assays such as competitive binding assays, thermal shift assays, or surface plasmon resonance (SPR), providing quantitative measurements like IC50 values or dissociation constants ($K_{D}$). Beyond direct binding, IEDB also curates T-cell epitope data where the presented peptide is known to activate CD8$^{+}$ T cells. Such datasets are crucial for training machine learning models to predict not only binding affinity but also the likelihood of a peptide being naturally processed and presented, forming the basis for immunoinformatics tools like NetMHCpan [53] and MHCflurry [54]. The diversity of peptide lengths (typically 8–11 amino acids) and HLA alleles in these datasets allows for broad applicability in identifying potential cytotoxic T-cell epitopes. Additionally, this field is augmented by other notable databases and datasets, including those derived from large-scale immunopeptidome profiling efforts [55, 56], as well as specialized repositories like EPIMHC [57], MHCBN [58], and Syfpeithi [59].

### Datasets for peptide–MHC class II binding

Similar to MHC class I, datasets for peptide–MHC class II binding are predominantly aggregated within the IEDB [33]. This repository contains a wealth of experimental measurements detailing the binding of larger peptides (typically 10–16 amino acids) to various MHC class II alleles (e.g. HLA-DR, -DQ, and -DP). The experimental methodologies for collecting these data often mirror those used for MHC class I, including SPR and competitive binding assays, yielding quantitative affinity data. Beyond these general resources, specialized datasets continue to emerge, such as those derived from HLA-II immunopeptidome profiling and deep learning efforts [60], which further reveal intricate features of antigenicity to inform antigen discovery. Notably, repertoire-scale determination of MHC II peptide binding via yeast display has also significantly improved antigen prediction by providing large-scale binding data [61]. It is also important to note that databases primarily focused on TCR–pMHC interactions, such as VDJdb [62], also implicitly or explicitly contain information on MHC class II presented peptides by detailing the specific pMHC II complexes recognized by their curated TCRs. These comprehensive datasets are indispensable for developing and validating computational models tailored for MHC class II peptide binding prediction, which is critical for identifying epitopes recognized by CD4$^{+}$ helper T cells. The continuous expansion of these datasets, covering more diverse human populations and pathogen-derived peptides, remains vital for improving the generalizability of predictive algorithms.

### Datasets for TCR–pMHC binding

Predicting the specific interaction between a TCR and its cognate pMHC complex poses unique data challenges due to the vast diversity of TCRs and the intricate nature of the tripartite binding interface. Key public resources for TCR–pMHC binding datasets include IEDB [33], VDJdb [62], McPAS-TCR [64], pan immune repertoire database (PIRD) [67], TetTCR-seq [23] and 10$\times $ Genomics [66]. VDJdb is a widely used database that curates experimentally validated TCR sequences (often $\beta $ chains, but increasingly paired $\alpha \beta $ chains) and their associated antigen specificities, primarily focusing on viral and bacterial antigens. McPAS-TCR offers a similar collection, specializing in pathogen- and disease-related TCR sequences linked to their specific epitopes and MHC restrictions. While these databases provide crucial insights into TCR clonotypes linked to known antigens, direct measurements of TCR–pMHC binding affinity are less common compared with MHC–peptide data. Emerging specialized datasets continue to enrich this field, often derived from high-throughput functional screens, single-cell TCR sequencing, and pMHC multimer sorting assays. Notable additions include TRAIT [71], a comprehensive database for TCR–antigen interactions. Data from studies like Heikkila *et al.* [68] and Dash *et al.* [65] provide insights into human thymic T-cell repertoires and define quantifiable predictive features for epitope-specific TCRs. Furthermore, large-scale initiatives like ImmuneCODE [70], an open-access database from The ImmunoRACE study, contribute extensive real-world immune response data to COVID-19 events. While primarily known for medicinal chemistry, BindingDB [63] also includes some relevant interactions that may be leveraged for TCR–pMHC studies. Besides, Zhou *et al.* developed NeoTCR [69], an immunoinformatic database of TCR sequences with neoantigen specificities. These growing and diverse datasets, despite being smaller in scale for direct TCR–pMHC binding affinity, are pivotal for training advanced AI models to decode the complex TCR-$\alpha \beta $-pMHC recognition landscape and to identify truly immunogenic epitopes.

It should be noted that although there is a degree of overlap between these datasets, each contributed a sufficient number of unique samples. The overall introduction to these datasets is presented in Table 1.

**Table 1:** Overview of common publicly available datasets for T-cell antigen identification.

**Dataset**	**Published in**	**Resources**
**Peptide–MHC I binding**		
SYFPEITHI [59]	Immunogenetics, 1999	http://www.syfpeithi.de/
MHCBN [58]	Bioinformatics, 2003	http://www.imtech.res.in/raghava/mhcbn
EPIMHC [57]	Bioinformatics, 2005	http://immunax.dfci.harvard.edu/bioinformatics/epimhc/
Abelin *et al*. [56]	Immunity, 2017	https://www.cell.com/immunity/fulltext/S1074-7613(17)30042-0∖#mmc1
IEDB [33]	Nucl. Acids Res., 2019	https://www.iedb.org/
Sarkizova *et al*. [55]	Nat. Biotechnol., 2020	https://massive.ucsd.edu/ProteoSAFe/static/massive.jsp?redirect=auth
**Peptide–MHC II binding**		
VDJdb [62]	Nucl. Acids Res., 2018	https://vdjdb.cdr3.net/
IEDB [33]	Nucl. Acids Res., 2019	https://www.iedb.org/
Rappazzo *et al*. [61]	Nat. Commun., 2020	https://www.nature.com/articles/s41467-020-18204-2∖#Sec24
Strazar *et al.* [60]	Immunity, 2023	https://www.cell.com/immunity/fulltext/S1074-7613(23)00226-1
**TCR–pMHC binding**		
BindingDB [63]	Nucl. Acids Res., 2016	https://www.bindingdb.org/
McPAS-TCR [64]	Bioinformatics, 2017	http://friedmanlab.weizmann.ac.il/McPAS-TCR/
Dash *et al.* [65]	Nature, 2017	https://www.ncbi.nlm.nih.gov/search/all/?term=SRP101659
VDJdb [62]	Nucl. Acids Res., 2018	https://vdjdb.cdr3.net/
TetTCR-seq [23]	Nat. Biotechnol., 2018	https://www.ncbi.nlm.nih.gov/gap/
IEDB [33]	Nucl. Acids Res., 2019	https://www.iedb.org/
10$\times $ [66]	Tech. Rep., 2019	https://www.10xgenomics.com/
PIRD [67]	Bioinformatics, 2020	https://db.cngb.org/pird/
Heilkkila *et al.* [68]	Mol. Immunol., 2020	https://www.ebi.ac.uk/ena/browser/view/PRJEB41936
NeoTCR [69]	Genomics Proteomics Bioinformatics, 2024	http://neotcrdb.bioxai.cn/home
ImmuneCODE [70]	Front. Immunol., 2025	https://clients.adaptivebiotech.com/pub/covid-2020
TRAIT [71]	Genomics Proteomics Bioinformatics, 2025	https://pgx.zju.edu.cn/traitdb/

### Negative sample generation strategies

In supervised machine learning for binding prediction, models require both positive (binding) and negative (nonbinding) samples for training. While positive samples are derived from experimental data, collecting sufficient high-quality negative samples is often challenging, as most biological experiments focus on identifying binders rather than nonbinders. Consequently, various strategies are employed to generate reliable negative samples. The strategies used differ significantly due to the inherent complexity of the interactions.

In peptide–MHC binding prediction, the generation of negative samples aims to create peptide–MHC pairs that are known or highly unlikely to bind, primarily leveraging the vastness of the peptide space and the known specificity of MHC alleles. Common strategies include randomly sampling (RS) peptides that are known not to bind or simply generating random peptide sequences, as the vast majority of random peptide–MHC combinations would not result in binding. Additionally, a highly effective and biologically relevant approach is to pair known binding peptides with noncognate MHC alleles (NC), exploiting the high polymorphism and exquisite specificity of MHC molecules to generate robust negative examples. More sophisticated methods in this category might also include decoy peptides, which are designed to be structurally or chemically similar to known binders but lack binding activity, thereby posing a greater challenge for the model’s discrimination.

**Figure 3:**
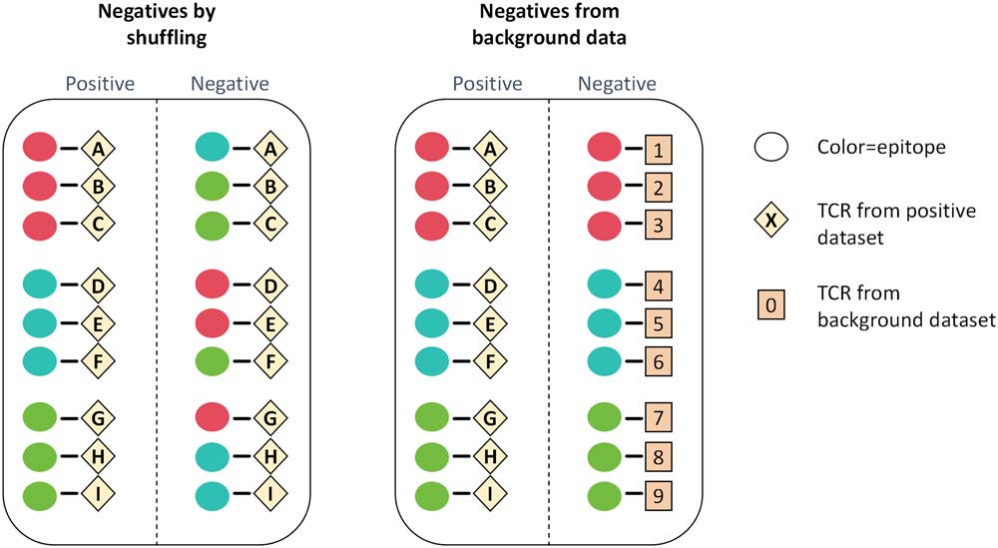
Negatives generation in TCR–pMHC task. The left panel demonstrates generating negatives through shuffling, where existing epitopes and TCRs are permuted such that each TCR is associated with a noncognate epitope. The right panel depicts the generation of negatives from a background dataset, involving the pairing of novel TCR sequences with known epitopes. Reproduced from [72] .

Conversely, negative sample generation in TCR–pMHC binding prediction is more intricate due to the complex tripartite interaction involving the TCR, peptide, and MHC. The core challenge lies in creating nonbinding TCR–pMHC triples that accurately reflect the immense noninteracting repertoire without being too easily distinguishable by the model; crucially, the pMHC component within these negative samples is often a valid, existing complex. As shown in Fig. 3, two primary strategies are employed: one involves “negatives by shuffling (SH)”, where experimentally validated TCRs are purposefully paired with different, noncognate pMHC complexes from the positive dataset, ensuring individual components are biologically real but their specific combination is known not to interact. The other strategy, “negatives from background data (BK)”, involves pairing known positive pMHC complexes with new TCR sequences randomly sampled from a general T-cell repertoire, effectively serving as background noise and reflecting the immense diversity of TCRs that typically do not bind to a given antigen.

## AI-driven approaches in T-cell antigen identification

The application of AI has transformed T-cell antigen identification by leveraging advanced machine learning (ML) and deep learning (DL) techniques to model complex molecular interactions. These AI-driven methods predict peptide–MHC binding and TCR–pMHC interactions, critical for developing effective mRNA vaccines and immunotherapies. Before delving into specific AI models, it is essential to understand how biological sequences are represented for computational analysis, which is crucial for model training and typically involves the generation of these representations.

### Amino acid sequence encoding for AI models

**Figure 4:**
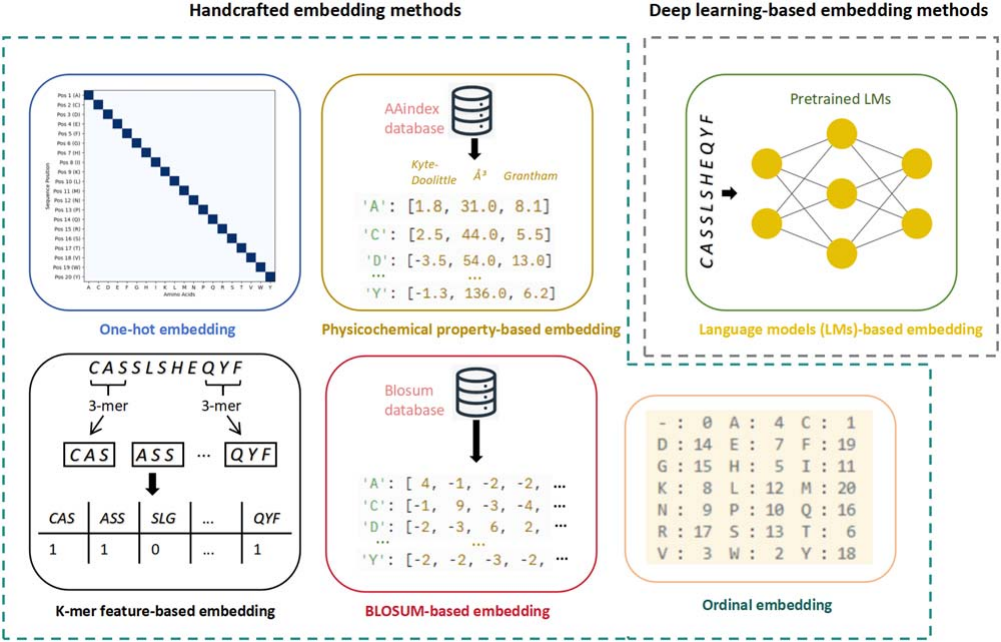
Overview of different peptide embedding methods for AI models. This figure categorizes peptide sequence embedding techniques into handcrafted embedding methods and deep learning-based embedding methods. Handcrafted methods include one-hot embedding, physicochemical property-based embedding, K-mer feature-based embedding, BLOSUM-based embedding, and ordinal embedding. Deep learning-based methods are primarily represented by language models (LMs)-based embedding utilizing pretrained LMs.

To enable AI models to process and learn from complex biological sequences, proteins—comprised of amino acid chains—must be effectively transformed into numerical representations. This crucial step, known as protein sequence embedding or feature representation, converts raw sequences into meaningful numerical vectors. As illustrated in Fig. 4, these methods can be broadly categorized into two main groups: handcrafted embedding methods and deep learning-based embedding methods [73].

Handcrafted embedding methods include ordinal embedding, one-hot embedding, physicochemical property-based embedding, K-mer feature-based embedding, and BLOSUM-based embedding approaches. Ordinal encoding (also known as integer encoding) assigns a unique integer to each distinct amino acid type. While simple and space-efficient, this method can inadvertently introduce false ordinal relationships between amino acids, potentially misleading models. More robust handcrafted methods include one-hot encoding, where each amino acid is represented by a unique binary vector (e.g. a “1” at one specific position and “0”s elsewhere in a 20D vector for the 20 standard amino acids), creating a sparse representation. Physicochemical property-based embedding leverages the intrinsic biochemical characteristics of amino acids, such as hydrophobicity, molecular weight, or charge. Each amino acid is mapped to a vector containing its intrinsic property values. These can be comprehensive, high-dimensional vectors sourced from databases like AAindex [74], or reduced representations like Atchley factors [75], which summarize key properties into a lower-dimensional vector. A notable challenge with such feature sets is the inherent subjectivity in selecting which properties or factor representations are most relevant for a specific prediction task, which can introduce variability and requires careful validation. K-mer feature-based embedding captures short, contiguous sequence patterns; for a given K (e.g. 3-mer), the sequence is broken down into overlapping K-mers, and the resulting embedding vector is then formed by the frequency or count of each possible K-mer within the sequence. Lastly, BLOSUM-based embedding [76] utilizes amino acid substitution matrices like BLOSUM50 or BLOSUM62 [77]. These matrices quantify the likelihood or score of one amino acid replacing another based on evolutionary conservation, providing representations that reflect biochemical similarity and evolutionary relatedness.

By contrast, deep learning-based embedding methods primarily refer to LM-based embedding. Akin to models in natural language processing (NLP), LMs are trained on vast unannotated protein sequence datasets to learn the complex “grammar” and “semantics” of protein sequences. Through this pretraining, these models learn to embed each amino acid or a subsequence into a dense, high-dimensional vector space. The embeddings are context-aware, meaning the representation of a particular amino acid can vary based on its surrounding amino acids in the sequence. These pretrained LMs (e.g. BERT [78, 79], ESM [80–82], and MSA Transformer [83]) offer highly informative embeddings that capture intricate structural and functional relationships, often outperforming handcrafted features in downstream predictive tasks like protein binding.

These diverse embedding strategies are foundational for applying AI models to tasks such as MHC–peptide binding prediction and TCR-pMHC binding prediction, as they enable the conversion of biological sequences into a format comprehensible and learnable by sophisticated algorithms.

Below, we outline the leading AI approaches for MHC class I–peptide binding, MHC class II–peptide binding, and TCR–pMHC binding, focusing on their technical architectures, unique innovations, and their roles in advancing antigen discovery.

### AI for MHC class I–peptide binding prediction

Predicting peptide binding to MHC class I molecules is the cornerstone for identifying cytotoxic T-cell epitopes. The application of AI in this field has evolved from classic neural networks to advanced deep learning architectures. As a foundational method, NetMHCpan-4.1 [53] employs an ensemble artificial neural network to predict binding affinity. Concurrently, other methods like Anthem [84] have explored variants of the Bayesian computational framework. Recent research has shown a clear shift towards more expressive architectures, particularly Transformers and attention mechanisms, to capture complex sequence contexts. For example, TransPHLA [85] introduced a Transformer-based architecture to optimize vaccine design. BigMHC [86] built a complex ensemble model fusing a wide LSTM, self-attention, and preattention blocks to learn intricate binding patterns. UnifyImmun [87] utilizes a cross-attention mechanism to process sequence embeddings of HLA, antigen, and TCR. Furthermore, another significant trend is the development of unified frameworks and the fusion of structural information. The UniPMT [88] framework represents the peptide–MHC–TCR relationship as a graph, using a graph neural network (GNN) to update embeddings. Similarly, deepAntigen [89] adopts a graph convolutional network to identify T-cell antigens at the atomic level. These advancements highlight the shift from single-sequence analysis to more complex architectures to improve the precision of MHC-I binding predictions.

### AI for MHC class II–peptide binding prediction

Predicting binding for MHC class II is more challenging than for class I due to its open-ended binding groove and greater peptide length variability. AI methods are therefore designed to decode these complex interactions, with a particular focus on identifying the variable “binding core.” To address this challenge, researchers have developed various deep learning architectures. DeepSeqPanII [90] employs a recurrent neural network (RNN) with an attention mechanism, using LSTM and attention blocks to predict binding affinity and identify the binding core. DeepMHCII [91] utilizes a custom “binding interaction convolutional layer” to process peptide sequences and MHC-II pseudo sequences. Other approaches enhance accuracy and robustness through multi-stage or ensemble strategies. MixMHC2pred2.0 [92] is a two-block neural network model that first learns MHC-II allele binding specificity and then combines it with sequence, length, and context features in a second block for final prediction. NetMHCIIpan4.2 [93] and NetMHCIIpan4.3 [94], as extensions of the NNAlign_MA [95] method, leverage a massive ensemble of 100 deep learning models and incorporate strategies like peptide inversion. Similar to its application in MHC-I prediction, deepAntigen [89] also demonstrates its versatility here, using a graph convolutional network to handle MHC-II binding. Nevertheless, fewer methods exist for MHC class II compared with class I due to these inherent complexities.

### AI for TCR–pMHC binding prediction

Predicting TCR–pMHC interactions is the most intricate task in T-cell antigen identification, as it involves the highly cooperative and precise interface of the TCR, peptide, and MHC. The evolution of AI models in this domain shows a clear progression from “feature concatenation” to “deep representation” and finally to “integrating prior knowledge.” Early AI models typically relied on feature concatenation and ensemble learning. For instance, ERGO-II [96] is an MLP-based model that predicts binding probability by concatenating encoded features of the TCR and peptide (generated via LSTM or autoencoders). DLpTCR [97] employs an ensemble deep learning model integrating FCN, LeNet-5, and ResNet. To more deeply capture intrinsic sequence relationships, subsequent models developed more complex embedding and architectural strategies. PMTnet [98] uses a stacked auto-encoder to learn deep embeddings for the TCR. Given the vast diversity of TCRs, recent models have begun to focus on solving the generalization problem, especially for predicting binding to unseen peptides. PanPep [99] adopts a framework combining meta-learning with a neural Turing machine, enabling it to adapt to new tasks quickly. The latest trend is the integration of physical and biological prior knowledge to enhance model accuracy and interpretability. The PISTE [100] method, a physics-inspired sliding transformer, integrates the gradient fields of amino acid interactions into its attention mechanism. This allows it to intelligently navigate biosequence interactions, improving generalization for TCR–peptide–HLA binding prediction, even for rare sequences.

It is worth noting that some methods, such as UnifyImmun [87] and UniPMT [88], are capable of performing both peptide–MHC class I binding prediction and TCR–pMHC binding prediction. Furthermore, deepAntigen [89] demonstrates even broader applicability, handling peptide–MHC class I, peptide–MHC class II, and TCR–pMHC binding predictions. The representative AI methods for T-cell antigen identification, including peptide–MHC I binding prediction, peptide–MHC II binding prediction, and TCR–pMHC binding prediction, as well as the used datasets, embedding methods, negative sample generation methods, evaluation metrics, and code resources, are shown in Table 2.

**Table 2:** Representative AI methods for T-cell antigen identification.

**Model**	**Published in**	**Datasets used** ^a^	**N.G.** ^b^	**E.M.** ^c^	**Metrics** ^d^	**Resources**
**Peptide–MHC I binding prediction**						
NetMHCpan-4.1 [53]	Nucl. Acids Res., 2020	IEDB [33]	RS	BL	AUROC PPVn	http://www.cbs.dtu.dk/services/NetMHCpan-4.1/
Anthem [84]	Brief. Bioinform., 2021	IEDB [33] EPIMHC [57] MHCBN [58] SYFPEITHI [59]	RS	BL	Sensitivity Specificity Accuracy MCC AUROC	https://github.com/17shutao/Anthem
TransPHLA [85]	Nat. Mach. Intell., 2022	IEDB [33] EPIMHC [57] MHCBN [58] SYFPEITHI [59]	RS	OR	Accuracy MCC F1-Score AUROC	https://github.com/a96123155/TransPHLA-AOMP
STMHCpan [101]	Brief. Bioinform., 2023	IEDB [33]	RS	OR	Recall Precision F1-Score Accuracy AUROC	https://github.com/Luckysoutheast/STMHCPan
MixMHCpred2.2 [86]	Cell Systems, 2023	Self-curated datasets from multiple public sources	RS	BL	AUROC PPV	https://github.com/GfellerLab/MixMHCpred/releases
BigMHC [102]	Nat. Mach. Intell., 2023	IEDB [33] NEPdb [103] TESLA [104] Neopepsee [105] MANAFEST [106]	RS	OH	AUROC AUPRC PPVn	https://github.com/KarchinLab/bigmhc
ImmuneApp [107]	Nat. Commun., 2024	Self-curated datasets from multiple public sources	RS	BL	AUROC AUPRC PPVn	https://github.com/bsml320/ImmuneApp
MixMHCpred3.0 [108]	Genome Med., 2025	Self-curated datasets from multiple public sources	RS	BL	AUROC AUPRC	https://github.com/GfellerLab/MixMHCpred
UniPMT [88]	Nat. Mach. Intell., 2025	IEDB [33] TESLA [104] NEPdb [103] Neopepsee [105] MANAFEST [106]	RS	LM	AUROC AUPRC	https://github.com/ethanmock/UniPMT
UnifyImmun [87]	Nat. Mach. Intell., 2025	IEDB [33] EPIMHC [57] MHCBN [58] SYFPEITHI [59]	RS & NC	OR	AUROC AUPRC Accuracy MCC F1-Score	https://github.com/hliulab/UnifyImmun
deepAntigen [89]	Nat. Commun., 2025	IEDB [33] Sarkizova *et al.* [55] Abelin *et al.* [56] TESLA [104] Xu *et al.* [107]	RS	OH	AUROC AUPRC Sensitivity Specificity Precision NPCC	https://github.com/JiangBioLab/deepAntigen
**Peptide–MHC II binding prediction**						
DeepSeqPanII [90]	IEEE/ACM Trans. Comput. Biol. Bioinform., 2021	IEDB [33]	Not mentioned	OH & BL	AUROC SRCC	https://github.com/pcpLiu/DeepSeqPanII
DeepMHCII [91]	Bioinformatics, 2022	IEDB [33]	Not mentioned	OR	AUROC PCC	https://github.com/yourh/DeepMHCII
MixMHC2pred2.0 [92]	Immunity, 2023	Self-curated datasets from multiple public sources	RS	OH & BL	AUROC	https://github.com/GfellerLab/MixMHC2pred
NetMHCIIpan4.2 [93]	Commun. Biol., 2023	Self-curated datasets from multiple public sources	RS	BL	AUROC PPVn	https://services.healthtech.dtu.dk/services/NetMHCIIpan-4.2/
NetMHCIIpan4.3 [94]	Sci. Adv., 2023	Self-curated datasets from multiple public sources	RS	BL	AUROC PPVn	https://services.healthtech.dtu.dk/services/NetMHCIIpan-4.3/
deepAntigen [89]	Nat. Commun., 2025	Rappazzo *et al.* [61] Strazar *et al.* [60]	RS	OH	AUROC AUPRC Sensitivity Specificity Precision NPCC	https://github.com/JiangBioLab/deepAntigen
**TCR–pMHC binding prediction**						
ERGO-II [96]	Front. Immunol., 2021	McPAS-TCR [64] VDJdb [62] Kanakry *et al.* [109]	SH	OH	AUROC	https://github.com/IdoSpringer/ERGO-II
NetTCR-2.0 [110]	Commun. Biol., 2021	IEDB [33] VDJdb [62] 10$\times $ [66] MIRA [111]	SH	PC	AUROC PPVn	https://services.healthtech.dtu.dk/services/NetTCR-2.0/
ImRex [112]	Brief. Bioinform., 2021	VDJdb [62] McPAS-TCR [64] Dean *et al.*[113]	BK & SH	PC & BL	AUROC AUPRC	https://github.com/pmoris/ImRex
DLpTCR [97]	Brief. Bioinform., 2021	TetTCR-seq [23] VDJdb [62] IEDB [33]	BK	OH & PC	Recall Precision Accuracy AUROC	https://github.com/jiangBiolab/DLpTCR
pMTnet [98]	Nat. Mach. Intell., 2021	PIRD [67] McPAS-TCR [64] VDJdb [62] 10$\times $ [66] TetTCR-seq [23] Chen *et al.* [114]	SH	BL	AUROC AUPRC	https://github.com/tianshilu/pMTnet
DeepTCR [115]	Nat. Commun., 2021	Dash *et al.* [65] 10$\times $ [66] Glanville *et al.* [116] ImmunoMap [117] Chan *et al.* [118]	Not mentioned	OH	AUROC Recall Precision F1-Score	https://github.com/sidhomj/DeepTCR
TITAN [119]	Bioinformatics, 2021	VDJdb [62] ImmuneCODE [70] BindingDB [63]	SH	BL	Accuracy AUROC	https://github.com/PaccMann/TITAN
PRIME2.0 [86]	Cell Systems, 2023	Self-curated datasets from multiple public sources	BK	BL	AUROC PPV	https://github.com/GfellerLab/PRIME
TEINet [120]	Brief. Bioinform., 2023	VDJdb [62] McPAS-TCR [64] Lu *et al.* [98]	SH & BK	LM	AUROC Accuracy Precision Recall	https://github.com/jiangdada1221/TEINet
PanPep [99]	Nat. Mach. Intell., 2023	IEDB [33] VDJdb [62] PIRD [67] McPAS-TCR [23] ImmuneCODE [70]	BK	PC	AUROC AUPRC	https://github.com/bm2-lab/PanPep
TEIM [121]	Nat. Mach. Intell., 2023	VDJdb [62] McPAS-TCR [64] ImmuneCODE [70] IEDB [33]	SH	BL	AUROC MCC AUPRC	https://github.com/pengxingang/TEIM
PISTE [100]	Nat. Mach. Intell., 2024	VDJdb [62] McPAS-TCR [64] Lu *et al.* [98]	SH & BK	OR	AUROC AUPRC PPVn	https://github.com/jychen01/PISTE
MixTCRpred [122]	Nat. Commun., 2024	VDJdb [62] McPAS-TCR [64] IEDB [33] 10$\times $ [66]	SH & BK	OR	AUROC	https://github.com/GfellerLab/MixTCRpred
TPepRet [123]	Bioinformatics, 2025	IEDB [33] VDJdb [62] McPAS-TCR [64]	SH	PC	AUROC AUPRC	https://github.com/CSUBioGroup/TPepRet
UniPMT [88]	Nat. Mach. Intell., 2025	TetTCR-seq [23] VDJdb [62] IEDB [33] McPAS-TCR [64] PIRD [67]	SH	LM	AUROC AUPRC	https://github.com/ethanmock/UniPMT
UnifyImmun [87]	Nat. Mach. Intell., 2025	TetTCR-seq [23] VDJdb [62] IEDB [33] PIRD [67] Heilkkila *et al.* [68] 10$\times $ [66] ImmuneCODE [70] BindingDB [63]	SH & BK	OR	AUROC AUPRC Accuracy MCC F1-Score	https://github.com/hliulab/UnifyImmun
TCRBagger [124]	Cell System, 2025	IEDB [33] VDJdb [62] McPAS-TCR [64] PIRD [67]	BK	PC	AUROC AUPRC	https://github.com/bm2-lab/TCRBagger
deepAntigen [89]	Nat. Commun., 2025	IEDB [33] VDJdb [62] McPAS-TCR [64] PIRD [67] ImmuneCODE [70] NeoTCR [69]	BK	OH	AUROC AUPRC Sensitivity Specificity Precision NPCC	https://github.com/JiangBioLab/deepAntigen

^a^Models marked with “self-curated datasets from multiple public sources” aggregate data from specific public repositories; details are provided in Supplementary Table S2.^b^N.G., Negatives generation; E.M., Embedding method; RS, Negatives by randomly sampling; NC, Negatives from noncognate MHC alleles; SH, Negatives by shuffling; BK, Negatives from background data.^c^OH, one-hot embedding; OR, ordinal embedding; PC, physicochemical property (or Atchley factors)-based embedding; KM, K-mer feature-based embedding; BL, BLOSUM-based embedding; LM, language model-based embedding.^d^The metrics used will be introduced in detail in Section “Evaluation metrics for T-cell antigen identification.”

## Evaluation metrics for T-cell antigen identification

To assess the performance of AI algorithms in predicting peptide–MHC and TCR–pMHC interactions, a range of evaluation metrics is used, primarily rooted in the assessment of binary classification. These metrics quantify how well a model discriminates between positive instances (e.g. binding pairs) and negative instances (e.g. nonbinding pairs), and they are crucial for comparing different algorithms. Commonly used metrics include:

Area under the receiver operating characteristic curve (AUROC or AUC): This metric evaluates the model’s ability to distinguish between positive and negative classes across all possible classification thresholds. An AUROC of 1.0 indicates a perfect classifier, while 0.5 suggests random prediction. It is often preferred when assessing the overall discriminative power of a model, especially when the positive and negative classes are relatively balanced. The ROC curve plots the true positive rate (TPR) against the false positive rate (FPR) at various threshold settings. The formulas for TPR and FPR are: 


(1) \begin{align*} & \text{TPR (Sensitivity or Recall)} = \frac{\mathrm{TP}}{\mathrm{TP} + \mathrm{FN}}, \end{align*}

(2) \begin{align*} & \mathrm{FPR} = \frac{\mathrm{FP}}{\mathrm{FP} + \mathrm{TN}}, \end{align*}

where true positive (TP) represents correctly predicted positive instances, and true negative (TN) represents correctly predicted negative instances. False positive (FP) indicates incorrectly predicted positive instances (Type I error), and false negative (FN) indicates incorrectly predicted negative instances (Type II error).

The area under the precision-recall curve (AUPRC or PR-AUC): This metric is particularly informative for imbalanced datasets, where the number of negative instances significantly outweighs the number of positive instances (common in biological binding data). AUPRC evaluates the trade-off between precision and recall at different thresholds. A higher AUPRC indicates better performance on the positive class. The PR curve plots Precision against Recall at various threshold settings. The formulas for Precision and Recall are: 


(3) \begin{align*} & \mathrm{Precision} = \frac{\mathrm{TP}}{\mathrm{TP} + \mathrm{FP}}, \end{align*}

(4) \begin{align*} & \mathrm{Recall} = \frac{\mathrm{TP}}{\mathrm{TP} + \mathrm{FN}}, \end{align*}

where Precision represents the proportion of correctly predicted positive instances among all instances predicted as positive, and Recall (Sensitivity) represents the proportion of correctly predicted positive instances among all actual positive instances (same as TPR).

Accuracy: This is the ratio of correctly predicted instances (both positive and negative) to the total number of instances. While straightforward, it can be misleading in highly imbalanced datasets. The formula for Accuracy is: 


(5) \begin{align*}& \mathrm{Accuracy} = \frac{\mathrm{TP} + \mathrm{TN}}{\mathrm{TP} + \mathrm{TN} + \mathrm{FP} + \mathrm{FN}},\end{align*}

F1-Score: This is the harmonic mean of Precision and Recall, providing a balanced measure of a model’s accuracy, especially useful when there is an uneven class distribution. The formula for F1-Score is: 


(6) \begin{align*}& \mathrm{F1-Score} = 2 \times \frac{\mathrm{Precision} \times \mathrm{Recall}}{\mathrm{Precision} + \mathrm{Recall}},\end{align*}

Negative Pearson Correlation Coefficient (NPCC): This metric is the negative of the Pearson Correlation Coefficient (r), used when models output continuous scores. It measures the linear correlation between predicted scores ($Y_{p}$) and true values ($Y_{t}$). Lower NPCC (i.e. higher positive r) indicates a stronger agreement. The formula for the Pearson Correlation Coefficient (PCC) is: 


(7) \begin{align*}& \mathrm{PCC} = \frac{n \sum (Y_{p_{i}} Y_{t_{i}}) - (\sum Y_{p_{i}})(\sum Y_{t_{i}})}{\sqrt{[n \sum Y_{p_{i}}^{2} - (\sum Y_{p_{i}})^{2}][n \sum Y_{t_{i}}^{2} - (\sum Y_{t_{i}})^{2}]}},\end{align*}

where *n* is the number of data points, $Y_{p_{i}}$ is the $i$th predicted value, and $Y_{t_{i}}$ is the $i$th true value.

Positive Predictive Value (PPV): This metric measures the proportion of positive test results that are true positives, indicating a test’s accuracy in identifying true cases among those testing positive. It is calculated as the ratio of true positives to the total number of positive predictions. 


(8) \begin{align*}& \mathrm{PPV} = \frac{\mathrm{TP}}{\mathrm{TP} + \mathrm{FP}},\end{align*}

If evaluating the precision of the model only within its top “n” most confident positive predictions, this metric turns into PPVn. 


(9) \begin{align*}& \mathrm{PPVn} = \frac{\mathrm{TPn}}{\mathrm{TPn} + \mathrm{FPn}},\end{align*}

Matthews Correlation Coefficient (MCC): This metric is a balanced measure of the quality of binary classifications, particularly useful when the classes are of very different sizes. It takes into account TP, TN, FP, and FN, providing a more reliable and robust score than accuracy or F1-score for imbalanced datasets. The MCC is formulated as follows: 


(10) \begin{align*}& \mathrm{MCC} = \frac{TP \times TN - FP \times FN}{\sqrt{(TP+FP)(TP+FN)(TN+FP)(TN+FN)}},\end{align*}

Spearman Rank Correlation Coefficient (SRCC): The SRCC, denoted as $\rho $, quantifies the correlation between the ranks of two variables. It is particularly useful for evaluating the ranking quality of continuous model outputs (e.g. probability scores) against true binary labels. The formula for SRCC is given by: 


(11) \begin{align*}& \mathrm{SRCC} = 1 - \frac{6 \sum d_{i}^{2}}{n(n^{2} - 1)}.\end{align*}

An SRCC of 1.0 indicates a perfect positive monotonic relationship, while 0 denotes no monotonic relationship, and −1 signifies a perfect negative monotonic relationship. The term $d_{i}$ denotes the difference between the ranks of the predicted scores and true labels for the $i$th data point. The variable $n$ represents the total number of data points in the dataset.

As presented in Table 2, while various metrics exist, AUROC and AUPRC are particularly emphasized for evaluating AI algorithms in MHC–peptide and TCR–pMHC binding predictions due to their distinct sensitivities to data distribution. AUROC provides a comprehensive assessment of a classifier’s discriminative ability across all possible thresholds, making it robust to class imbalance in terms of its interpretation of overall ranking performance. However, in scenarios with highly imbalanced datasets, where the number of negative samples significantly outnumbers positive ones (a common occurrence in biological binding data where most random peptides do not bind), AUROC can sometimes provide an overly optimistic view of performance. This is because a classifier can achieve a high AUROC by correctly identifying a large number of true negatives, even if its performance on the minority positive class is modest.

Conversely, AUPRC focuses specifically on the positive class and is highly sensitive to the balance between precision and recall. It is less affected by a large number of true negatives and provides a more realistic assessment of a model’s ability to identify actual binders, which are often the minority class of interest in antigen prediction. Therefore, a high AUPRC directly reflects a model’s effectiveness in identifying relevant positive instances without being diluted by the abundance of negative samples. Given these complementary strengths, it is highly recommended to present both AUROC and AUPRC when evaluating AI models for T-cell antigen identification. Using both metrics offers a more complete and nuanced understanding of algorithm performance, ensuring that models are not only good at distinguishing between classes generally but also excel at precisely identifying the critical, often rare, binding events.

## Benchmarking 18 TCR–pMHC binding predictors

The rapid proliferation of computational models for predicting TCR binding to pMHC, encompassing methodologies ranging from classical machine learning to advanced deep learning architectures (summarized in Table 2), underscores the field’s technological advancements. However, a significant scientific challenge persists: the absence of a standardized, community-wide benchmarking framework. Published performance metrics often suffer from high heterogeneity due to inconsistent data splits, varied negative sampling strategies, various tricks used in data input encoding, and reliance on internal or proprietary evaluation sets. This lack of standardized conditions renders direct, cross-publication comparisons of model efficacy unreliable, impeding researchers’ ability to accurately assess true predictive power, and identify robust methods.

Furthermore, the challenge of reproducibility is compounded by practical obstacles within the computational community. Many cutting-edge predictors lack readily accessible training code, or their implementations are rigidly encapsulated, presenting a steep learning curve for researchers, particularly novices entering this complex domain. To simultaneously address the need for robust scientific comparison and foster rapid community adoption, our benchmark is uniquely accompanied by a unified, modular, and extensively documented codebase. This standardized platform not only ensures the reproducibility of all results presented here but also provides researchers with a practical starting point for rapid method iteration and development.

### Model re-implementation and evaluation framework

To provide a much-needed, unified assessment, and establish a reproducible benchmark for the field, we conduct a comprehensive, head-to-head evaluation of 18 SOTA TCR–pMHC binding prediction methods described in Table 2: ERGO-II [96], NetTCR-2.0 [110], ImRex [112], DLpTCR [97], pMTnet [98], DeepTCR [115], TITAN [119], PRIME2.0 [86], TEINet [120], PanPep [99], TEIM [121], PISTE [100], MixTCRpred [122], TPepRet [123], UniPMT [88], UnifyImmun [87], TCRBagger [124], and DeepAntigen [89].

All models are re-implemented in PyTorch. To enable fair comparison, we apply a consistent evaluation framework with uniform data preprocessing and training parameters: 40 epochs, batch size of 64, Adam optimizer with learning rate $2\times 10^{-4}$ and weight decay $10^{-5}$ on McPAS-TCR dataset; 80 epochs, batch size of 64, Adam optimizer with learning rate $10^{-4}$ and weight decay $10^{-5}$ on IEDB and VDJdb datasets. While we strive to preserve original performance, we cannot guarantee 100% replication due to framework differences and implementation variations.

For practical accessibility and reproducibility, we note that the computational resource requirements for all evaluated models remain modest. We successfully reproduced and benchmarked all 18 models on a single server equipped with a 15 GB Tesla T4 GPU. Under our specified training parameters (e.g. a batch size of 64), the peak GPU memory consumption for any single method did not exceed 5 GB. This demonstrates the high accessibility and ease of deployment of current T-cell antigen prediction algorithms.

### Implementation details and hyperparameter settings

To ensure methodological transparency and reproducibility, we provide detailed descriptions of our re-implementation approach for the 18 models listed above:

**(1) Hyperparameter determination.** All hyperparameters (e.g. number of layers, hidden dimensions, and attention heads) are determined following a consistent protocol. For models with publicly available source code (e.g. DeepTCR [115], ERGO-II [96], NetTCR-2.0 [110], ImRex [112], TEIM [121], MixTCRpred [122], PISTE [100], TPepRet [123], TCRBagger [124], and TEINet [120]), we directly adopt the architectures and hyperparameters described in the original papers or repositories. For models without open-source implementations or with insufficient architectural details (e.g. pMTnet [98], UniPMT [88], and PanPep [99]), we infer the network structure from the figures and descriptions in the respective papers, and validate them on small-scale data to ensure functional correctness. No model-specific hyperparameter tuning is performed to ensure a fair comparison across all methods.

**(2) Feature representation.** Amino acid sequences are encoded using one-hot representation (20 standard amino acids + padding). The maximum sequence lengths are set to the median lengths in the dataset (20 for CDR3$\beta $ and 15 for epitope); shorter sequences are padded, longer ones truncated. Categorical features (V/J genes and MHC) are one-hot encoded and projected through trainable linear embeddings. No external pretrained LMs (e.g. ProtBERT [125] and ESM [80–82]) or pretrained embeddings are used. For models that originally employ biochemical feature matrices (e.g. pMTnet [98], ImRex [112], and PanPep [99]), we use publicly available fixed matrices (Atchley factors or BLOSUM62) without fine-tuning.

**(3) Reproducibility.** To ensure full transparency, the complete PyTorch implementations of all 18 models, data preprocessing scripts, and training/evaluation pipelines are publicly available on GitHub. Supplementary Table S1 provides a detailed summary of architectural hyperparameters for each model. All experimental results (prediction probabilities, performance metrics, and ROC/PR curve data) are saved and publicly accessible.

### Comprehensive evaluation on public datasets

**Figure 5:**
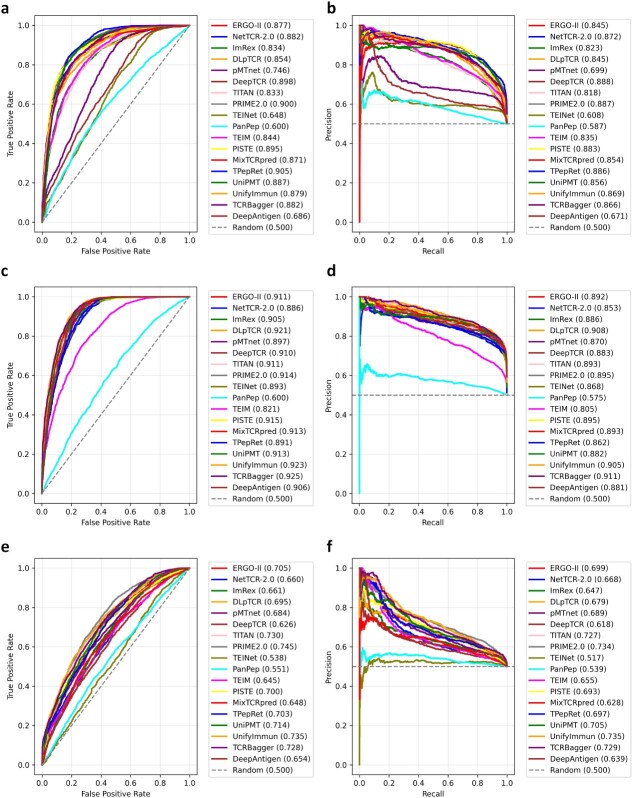
Performance of AUROC (a,c,e) and AUPRC (b,d,f) curves for all 18 re-implemented TCR-pMHC binding models across the three datasets: IEDB (top row), McPAS-TCR (middle row), and VDJdb (bottom row). Each curve is labeled with the model name followed by its corresponding metric value in parentheses. The dashed gray line represents the random baseline. Models are color-coded consistently across panels for comparability.

Evaluations are performed on three major public databases: the IEDB [33], McPAS-TCR [64], and VDJdb [62]. IEDB provides the largest volume of general binding pairs, VDJdb offers greater diversity in full-length TCR sequences, and McPAS-TCR focuses primarily on high-affinity, biologically confirmed interactions. Only TCR $\beta $-chain information (CDR3_beta, V_beta, and J_beta) is used, consistent with many models’ focus on $\beta $-chain dominance in MHC-I predictions. The negative binding pairs are generated by mismatching TCRs and pMHCs (via random permutation of TCR and pMHC components) within the same dataset while ensuring no overlap with positive pairs and preserving MHC restriction. A 1:1 positive-to-negative ratio is adopted to establish a balanced binary classification task, which is a standard and stable setting for initial comparative benchmarking of model architectures. We acknowledge that exploring biologically informed ratios is an important direction for future work aimed at clinical translation, as discussed in Section “Discussion.” Data are split into training (80%) and testing (20%) sets with stratified sampling to maintain class balance. Performance is quantified using metrics robust to classification imbalance: the AUROC and AUPRC, which provide a balanced measure even with highly skewed class sizes. The overall performance comparison is shown in Fig. 5.

#### Comparative findings

Our systematic benchmark reveals several critical insights regarding model performance, underscoring the influence of architectural designs, data encoding strategies, and dataset properties on predictive accuracy and generalization (see Fig. 5 for AUROC/AUPRC curves). Consistent with community-wide benchmarking efforts, we observe that Transformer- and Attention-based architectures (e.g. PRIME2.0 and UnifyImmun) generally excel in capturing long-range dependencies and handling sequence context, though they can be computationally intensive and sensitive to hyperparameter tuning. Convolutional or recurrent models (e.g. NetTCR-2.0 and DeepTCR) offer efficiency and simplicity but often struggle with sparsity, leading to inconsistent generalization. Ensemble methods (e.g. DLpTCR and TCRBagger) enhance stability through averaging but increase runtime without always yielding proportional gains in precision. Retrieval-augmented approaches (e.g. TPepRet) provide advantages in low-data scenarios by leveraging external embeddings but falter if database dimensions misalign with the model, potentially requiring additional projection layers. Notably, discrepancies with original papers—such as PanPep’s underperformance here versus its reported benchmarks—may stem from differences in negative sample generation: while the original often uses background database sampling (BK method) for more biologically realistic negatives, our unified shuffling (SH method) ensures fair comparison but potentially introduces harder or less representative examples, highlighting the sensitivity of models to negative data quality. Similarly, DeepAntigen’s lower performance compared with its original implementation may arise from the absence of atomic-level structural inputs used in our implementation, as our sequence-only framework limits its ability to model 3D interactions.

Data encoding significantly modulates these outcomes. Our uniform one-hot encoding for sequences and attributes (V/J genes, MHC) is computationally simple and interpretable, enabling direct comparability, but generates high-dimensional inputs (e.g. up to 7318 on VDJdb), increasing overfitting risk and memory demands while potentially diluting subtle physicochemical signals. Alternatives like Atchley factors (in pMTnet) or BLOSUM matrices compress representations to capture amino acid similarities, often improving generalization on sparse data by reducing dimensionality, though they may require additional projection layers and could overlook sequence-specific nuances in focused datasets. For optimal results, hybrid encodings—combining one-hot for hypervariable regions (e.g. CDR3) with learned embeddings for attributes—could balance expressiveness and efficiency, as evidenced by PRIME2.0’s success with pretrained features.

The bounds of generalization are starkly apparent across datasets, each amplifying specific model strengths and weaknesses:

- **IEDB (high volume but heterogeneous noise):** As the largest repository of binding pairs, IEDB yields solid mid-range performance, with PRIME2.0 (AUROC 0.900 and AUPRC 0.887) and TPepRet (AUROC 0.905 and AUPRC 0.886) leading, while simpler models like TEINet (AUROC 0.648 and AUPRC 0.608) falter amid noise. The dataset’s breadth favors Transformers that abstract global patterns, but one-hot’s high dimensionality exacerbates sensitivity to experimental variability, suggesting preprocessing (e.g. confidence filtering) as a key enhancement.
- **McPAS-TCR (focused high-affinity interactions):** This curated set elicits the strongest overall results, with PRIME2.0 (AUROC 0.914 and AUPRC 0.895) and ERGO-II (AUROC 0.911 and AUPRC 0.892) excelling due to its emphasis on validated, motif-rich pairs. The thematic focus benefits models capturing affinity rules, but even here, ensemble approaches like TCRBagger show gains in precision. One-hot encoding thrives in this low-sparsity environment, yet physicochemical alternatives might further refine affinity discrimination.
- **VDJdb (diverse but sparse full-length sequences):** Posing the toughest test, VDJdb induces widespread drops (e.g. PRIME2.0 at AUROC 0.745 and AUPRC 0.734; DeepTCR at AUROC 0.626 and AUPRC 0.618), exposing memorization in localized encoders amid its sparsity (many epitopes with <50 TCRs). Attention models (e.g. PRIME2.0 and UnifyImmun) hold relative advantages by modeling inter-sequence relations, but low AUPRC underscores the need for epitope-specific adaptations or advanced negatives to counter ambiguity in diverse repertoires.

In summary, this evaluation demonstrates that architectural choices like Transformers provide strong generalization for diverse data but demand careful encoding to manage dimensionality, while simpler models offer efficiency yet falter on sparsity. Dataset traits dictate success—IEDB for volume-driven robustness, McPAS for affinity precision, and VDJdb for generalization rigor—while encoding choices trade simplicity for expressivity. For future practitioners, we advise: (i) prioritizing per-epitope training on sparse sets like VDJdb to curb bias; (ii) experimenting with hybrid encodings to mitigate high-dimensional pitfalls; (iii) incorporating $\alpha $-chain data for fuller TCR context when available; (iv) exploring BK negative sampling alongside SH for sensitivity analysis; and (v) employing adaptive hyperparameters (e.g. lower lr for diverse data) with schedulers to ensure convergence. These recommendations aim to foster more reliable, transferable TCR prediction frameworks.

### Evaluation of model generalization on unseen epitope variant datasets

**Figure 6:**
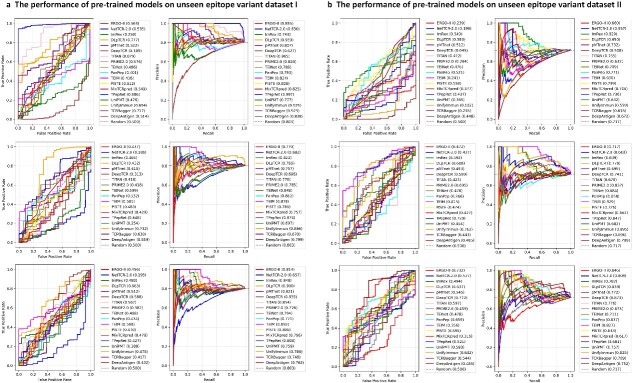
Generalization performance of pretrained TCR-pMHC models on two unseen epitope variant datasets. The figure plots AUROC and AUPRC curves for the 18 benchmarked models, evaluated on two OOD datasets: Unseen Epitope Variant Dataset I (a) and Unseen Epitope Variant Dataset II (b). Each row corresponds to the source dataset used for pretraining: IEDB (top), McPAS-TCR (middle), and VDJdb (bottom). Within each panel, the dashed gray line represents the random baseline (AUROC = 0.500). For the AUPRC plots, this baseline (also dashed gray) is set to the positive class proportion ($P$) of the respective test set: $P_{\text{ I}} = 0.803$ for Dataset I and $P_{\mathrm{II}} = 0.717$ for Dataset II, highlighting the challenging, high-positive-skew of the test data [126].

To assess the clinical utility and robustness of the benchmarked methods, we conduct a critical OOD generalization test on two independent Unseen Epitope Variant Datasets (Datasets I and II). These datasets, referenced from the stringent ePytope-TCR benchmark [126], are specifically designed to challenge models with novel peptide sequences, mirroring the difficulties encountered in real-world contexts where neoantigens frequently arise. The comprehensive performance evaluation is presented in Fig. 6.

The experimental design for these OOD tests imposes a dual challenge: (I) The negative samples are not derived directly from the original datasets (which are typically highly positive-skewed or lack sufficient high-quality negatives). Instead, they are artificially generated using a stringent MHC-constrained shuffling strategy, a variant of the SH strategy. It ensures that within a negative TCR–pMHC triple, the pMHC complex is a real, known binder (often from the training set), but it is specifically paired with a TCR that is restricted to a different MHC allele. This creates a biologically plausible “hard negative” where the individual components are valid, but their specific combination is known not to interact due to MHC restriction mismatch. (II) The resulting evaluation sets exhibit high positive class proportions: the proportion is $P_{\mathrm{I}} = \mathbf{0.717}$ for Dataset I and $P_{\mathrm{II}} = \mathbf{0.803}$ for Dataset II. This design simulates a highly challenging biological scenario where the majority of tested pairs are positive. Consequently, the AUPRC random baseline is mathematically established at these high $P$ values ($0.803$ and $0.717$, respectively).

This high-baseline composition creates a scenario where models must demonstrate a predictive capability significantly exceeding this high $P$ value to be considered genuinely effective. Performance only slightly above the baseline represents minimal added value beyond simply predicting the majority positive class. Overall, the results confirm that the performance of the majority of predictors drops significantly when they transition from internal cross-validation to this external, highly challenging OOD setting. This finding is highly consistent with the conclusions drawn by the ePytope-TCR benchmark itself [41, 126].

A detailed examination of the AUPRC values (Fig. 6, the second and fourth columns) reveals the difficulty imposed by the high baselines and underscores the poor generalization of all models. On Dataset I (baseline $P_{\mathrm{I}} = 0.803$), the top-performing model (TPepRet trained on IEDB) achieves an AUPRC of 0.997. This represents a performance gain of $\approx $ 0.194 over an already extremely high baseline. On Dataset II (baseline $P_{\mathrm{II}} = 0.717$), the challenge is even more pronounced. The top-performing model (UnifyImmun trained on McPAS–TCR) achieves an AUPRC of 0.895. This gain of $\approx $ 0.178 highlights that even the best models struggle to significantly separate true positives in a challenging setting. Crucially, across both datasets, the vast majority of models—particularly those trained on VDJdb—fail to provide a meaningful gain over the random baselines, with many performing at or below them. These minimal gains underscore a critical finding: current models lack the necessary sophistication to reliably identify true positives when distinguishing between highly similar binding and nonbinding pairs.

- **Models trained on IEDB (Fig. 6, top row):** These models consistently demonstrate the most robust generalization. They achieve the highest overall AUROC and AUPRC scores, particularly dominating on Dataset I, and significantly surpass the high AUPRC baselines. This meaningful performance stems from the size and compositional heterogeneity of IEDB, which likely forces models to learn more universal rules governing TCR–pMHC interaction.
- **Models trained on McPAS-TCR (Fig. 6, middle row):** These predictors exhibit more mixed generalization performance. While generally trailing the IEDB-trained models, this group produced the top-performing model (UnifyImmun) on Dataset II, while still largely outperforming the models trained on VDJdb.
- **Models trained on VDJdb (Fig. 6, bottom row):** These models consistently demonstrate the weakest generalization. Many models struggle to meaningfully surpass the high AUPRC baselines, with some performing at or near the level of a random classifier. This suggests that training on the sparser, less diverse VDJdb dataset is insufficient for learning generalizable features for OOD prediction.

In conclusion, our evaluation of OOD strongly corroborates the existing literature by confirming a significant generalization gap between current TCR predictors, particularly when models are trained on smaller or less diverse datasets. In addition, it demonstrates that the use of large compositionally heterogeneous datasets such as IEDB is paramount to the development of models that effectively generalize to unseen epitope variants, a necessary step toward achieving actionable prediction in immunoinformatics.

## Discussion and future perspectives in AI-driven T-cell antigen identification

### Discussion

The encoding methods chosen significantly influence the effectiveness of AI models in the identification of T cell antigens [73, 119] for biological sequences and strategies for the generation of negative samples [72, 120]. Different embedding methods for TCRs and epitopes, as explored in recent benchmarks, possess distinct advantages and disadvantages. Handcrafted embedding methods, such as one-hot, physicochemical property-based, K-mer feature-based, BLOSUM-based, and ordinal encoding, provide interpretable features but may lack the ability to capture complex, context-dependent relationships within sequences. In contrast, deep learning-based embeddings, particularly those derived from LMs, offer context-aware, dense vector representations that can capture intricate structural and functional relationships, often outperforming handcrafted features in predictive tasks. Given the rapid proliferation of pretrained protein LMs [127–129], LM-based embeddings hold immense promise for future advancements in T-cell antigen identification.

Similarly, the choice of negative sample generation strategies is crucial, as biases in negative data can significantly impact model performance and generalization [72, 130]. For pMHC binding prediction, **RS** is a commonly employed strategy. It is well known that the **RS** strategy can potentially generate false negatives, despite methods utilizing **NC** offering superior biological relevance and robustness. In particular, as observed in Table 2, **NC**-based negative data generation methods are rarely, if ever, adopted by representative models for the prediction of pMHC class I and class II binding, in stark contrast to the widespread use of **RS**. This significant disparity warrants further investigation into the relative merits and challenges of **NC**-based methods. Future work should systematically compare **RS** and **NC** strategies, potentially employing cross-validation between these different negative sample generation approaches, to mitigate potential biases and enhance model robustness. For the TCR–pMHC interactions, **SH** and **BK** are predominantly used, each with implications for the robustness of the model and biological relevance [120]. Nevertheless, exploring more sophisticated negative sample generation strategies, particularly learning-based generative approaches such as generative adversarial networks [131], diffusion models [132], and autoregressive transformer [133], represents a promising direction for future research. Besides, exploring biologically informed ratios of positive to negative TCR–peptide pairs is also an important direction for future work aimed at clinical translation.

Another critical point for discussion is the ongoing challenge of data quality and quantity. The rigorous OOD evaluation we conduct in Section “Benchmarking 18 TCR–pMHC binding predictors” (Fig. 6) reveals a fundamental flaw in current TCR–pMHC prediction models: all models, regardless of their architecture or training data sources, experience a sharp decline in predictive performance when face with epitope variants that are not seen during training. This widespread OOD generalization failure suggests that current AI models (including complex attention-based architectures) may not have learned the “universal grammar” or potential biophysical rules for TCR–pMHC recognition. On the contrary, they are more likely to be highly specific “shortcut patterns” or overfitting to common TCR–epitope pairs in learning training data that are associated with specific epitopes. When there is a slight variation in the epitope sequence (such as in the OOD test set), these models lose their recognition ability. This discovery has significant implications for this field. For clinical applications such as the prediction of neoantigens or the development of TCR-T therapies, the ability of models to predict new epitopes is at the core of their value. Our benchmark testing confirms that the current model has failed at this point, which explains why the success rate of calculating predicted T-cell antigens in subsequent wet experiments is extremely low. Therefore, solving the generalization problem of OOD should be regarded as the primary challenge for the future development of computational methodology in the field of T-cell antigen recognition. It is crucial to note that the pronounced generalization gap identified here pertains specifically to the challenge of generalizing to sequence variants of known antigens/epitopes. This is a critical but not exhaustive test of OOD robustness. Future benchmarks must expand to evaluate generalization across truly novel antigens, diverse MHC alleles, and heterologous TCR repertoires, which represent even greater challenges for clinical deployment. Our work thus defines a clear and immediate frontier for methodological improvement, while acknowledging the broader landscape of generalization that lies ahead. In addition, our data show (Fig. 6, top row) that using the largest and most heterogeneous dataset like IEDB for training can lead to optimal relative generalization performance, emphasizing the need for more comprehensive, and diverse datasets in the future. However, relying solely on data expansion may not be enough, which once again confirms our viewpoint in Section “Future perspectives” that there is an urgent need for AI architectures that can integrate structural information (such as attempts at deepAntigen [89]), multimodal data, and more powerful protein LMs in the future, to capture universal binding patterns across different epitopes fundamentally.

### Future perspectives

**Figure 7:**
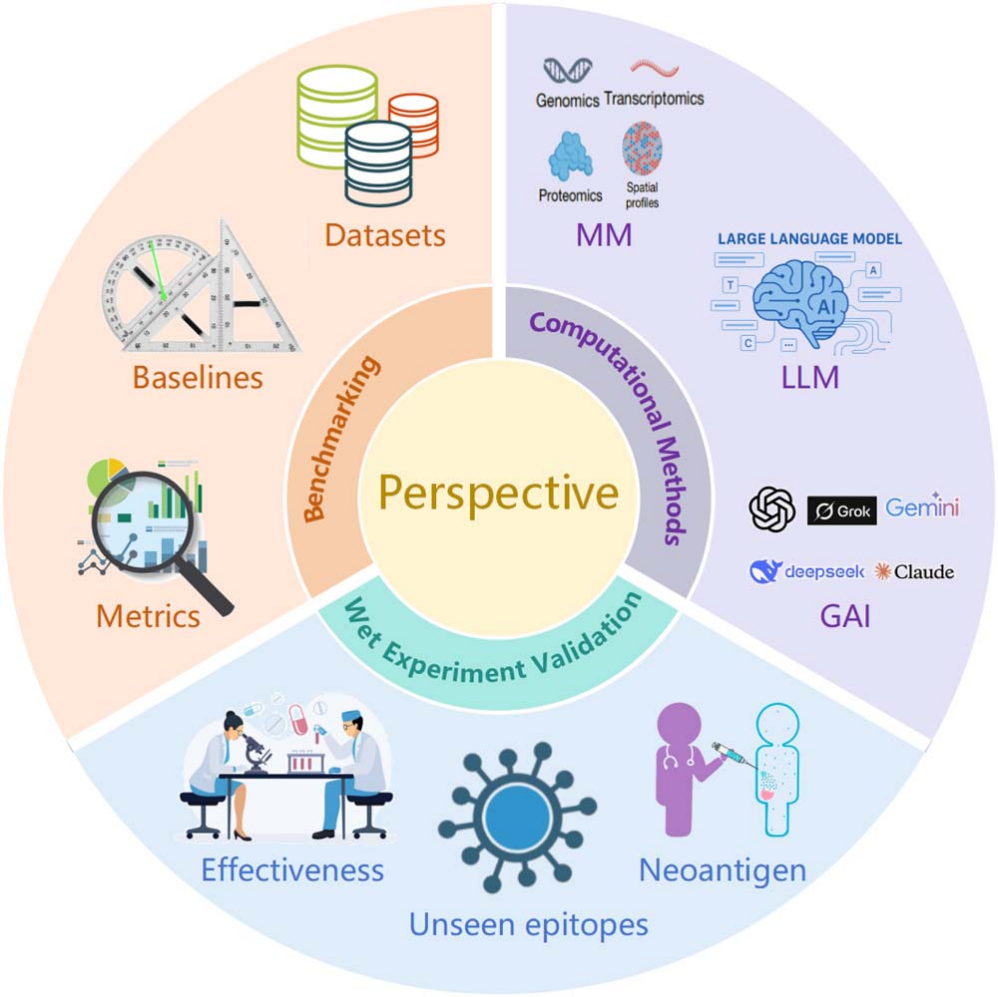
Conceptual framework for future perspectives in AI-driven T-cell antigen identification. This diagram illustrates three interconnected pillars—benchmarking, computational methods, and wet experiment validation—each comprising specific subareas crucial for advancing the field. Benchmarking encompasses datasets, baselines, and metrics; computational methods include multimodality (MM), large language models (LLM), and generative artificial intelligence (GAI); and wet experiment validation focuses on effectiveness, unseen epitopes, and neoantigens.

Future advancements in AI-driven T-cell antigen identification will be propelled by progress across three key areas: benchmarking, computational methods, and wet experiment validation, as illustrated in Fig. 7.

Just as ImageNet [134] has driven the historical process of AI, benchmarking will remain crucial for the rigorous evaluation and comparison of AI algorithms. Currently, the field faces a challenge where many methods utilize inconsistent datasets and do not consistently compare against SOTA methods, which often diminishes the persuasiveness of new approaches [35]. Therefore, establishing more unified public datasets with high quality and quantity, similar to MNIST [135] or ImageNet [134] dataset in the computer vision community and the MSD datasets [136] for medical image segmentation, is urgently needed to overcome current limitations such as inconsistent measurements and biases [126]. Beyond unification, it is crucial to further enrich data diversity, particularly by addressing the skewed distribution prevalent in existing datasets, which are often heavily concentrated on common alleles and epitopes while lacking sufficient data for rare alleles and those exhibiting a “long-tail” distribution [35]. This imbalance significantly limits the generalizability and robustness of AI models. There are two possible solutions to this issue: (i) establishing international consortia dedicated to profiling HLA alleles and TCR repertoires in underrepresented populations; and (ii) promoting the adoption of privacy-preserving federated learning frameworks to collaboratively train models on distributed clinical datasets without sharing raw data. Additionally, defining robust baselines by organizing open competitions, such as CASP [137] or CACHE [138], challenges, will help contextualize the performance of new models. Although we benchmark some existing TCR–pMHC models in this review, it is still crucial to establish a more comprehensive and systematic benchmarking. Notably, some work is currently underway, as exemplified by Wu *et al.* [139] in the context of epitope-HLA I binding and Drost *et al.* [126] in the context of TCR-epitope binding. The emergence of more bench work is expected to occur. Furthermore, a comprehensive set of metrics (including AUROC, AUPRC, Accuracy, F1-Score, NPCC, and PPV) will be essential for a nuanced understanding of model capabilities, especially in the context of imbalanced biological data.

**Figure 8:**
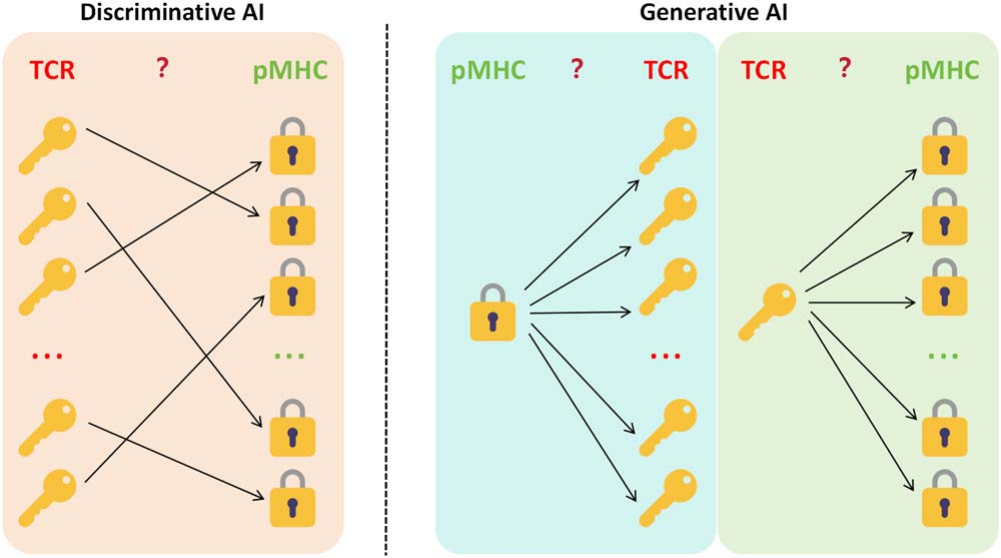
Illustrative comparison of discriminative and GAI for TCR–pMHC binding. The left panel shows discriminative AI, which predicts whether a given TCR (“key”) binds to a specific pMHC (“lock”). The right panel depicts GAI, which can either synthesize novel TCRs (“keys”) for a given pMHC (“lock”), or generate new pMHCs (“locks”) that are likely to be recognized by a specific TCR (“key”).

Computational methods will continue to evolve, with a focus on addressing the complexities of antigen recognition. MM will be key [140, 141], involving the development of AI models that can simultaneously process various types of data, such as genomic, proteomic, spatial transcriptomic, and structural information, learning complex interdependencies between different stages of antigen presentation and recognition. For example, current existing public structural TCR–pMHC datasets [142–145] and methods [89, 145, 146] provide inspiration and prospects, with which it is expected to fuse TCR sequence information and structure information to achieve more accurate T-cell antigen recognition. However, the primary bottleneck remains the extreme scarcity of high-resolution experimental structures for TCR–pMHC complexes, which limits the training data for structure-aware models. While computational methods like AlphaFold-Multimer [147] show promise, their accuracy for predicting the precise conformational changes upon TCR binding—critical for specificity—is still evolving and requires further benchmarking in this specific context. Beyond sequences and structures, integrating functional immune signatures—such as gene expression profiles of antigen-specific T cells from single-cell RNA-seq—could allow models to predict not just binding, but also the functional state and potential efficacy of the T-cell response, directly addressing the challenge of distinguishing tumor-reactive from bystander T cells [19]. In addition, the integration of LLMs, pretrained on vast protein sequence datasets, is expected to yield highly informative and context-aware embeddings [73], akin to their success in NLP. Currently, there are a large number of unlabeled TCR datasets such as TCRdb [148] and TCR [149–151] or MHC LMs [152–154]. Using these conditions, we are expected to develop TCR characterizers with better performance. Finally, the emergence of GAI [155–157] will open new avenues for antigen discovery. The application of GAI holds significant promise in this field [158–160]. As depicted in Fig. 8, while discriminative AI models focus on predicting the interaction between a given TCR and pMHC (analogous to a key fitting a specific lock), GAI offers a transformative capability. It can, for instance, take a specific pMHC (a “lock”) and generate novel TCR sequences (new “keys”) that are predicted to bind to it. Conversely, given a specific TCR (a “key”), GAI could propose new pMHC complexes (new “locks”) that this TCR is likely to recognize. This bidirectional generative capacity could revolutionize antigen discovery by enabling the *de novo* design of immunogenic peptides or therapeutic TCRs, moving beyond prediction to creation. Beyond generating novel sequences, a key advantage of GAI in this context is its capacity to systematically explore the vast, uncharted regions of the TCR–pMHC interaction space, potentially discovering high-affinity binders with noncanonical motifs that discriminative models, focused on the existing data, might overlook. It needs to be clarified that for clinical translation, inference speed of computational methods is generally not the primary bottleneck. Once trained, even complex models can process a patient’s candidate neoantigens (typically thousands) in minutes. The greater challenges remain prediction accuracy (especially OOD generalization) and the integration of AI into streamlined, automated clinical bioinformatics pipelines.

Wet experiment validation is indispensable for bridging the gap between *in silico* predictions and real-world biological outcomes. Focusing on effectiveness means moving beyond the simple prediction of binding to accurately predict functional immunogenicity, i.e. whether a predicted antigen elicits a robust and protective T-cell response *in vivo*. The concept of lab-in-the-loop is crucial here, as it facilitates an iterative process where computational predictions guide experimental validation, and experimental results, in turn, refine the computational models [140]. A minimal viable “lab-in-the-loop” framework could involve: (i) *in silico* screening of candidate neoantigen–TCR pairs using AI models; (ii) high-throughput *in vitro* validation using pMHC multimer staining or T-cell activation assays; (iii) retraining the AI model with the newly generated positive/negative data to refine its predictions for the next iteration, closing the loop between computation and experiment. The identification of unseen epitopes and neoantigens derived from tumor proteins is particularly critical for cancer immunotherapy and personalized medicine like TCR-T immunotherapy [161], requiring rigorous experimental validation to confirm their immunogenicity.

## Conclusion

This review systematically explores the evolving landscape of AI technologies in T-cell antigen identification, a critical step for the development of highly effective mRNA vaccines and other immunotherapies. The paper first delineates the intricate multi-step process of T-cell antigen recognition, highlighting the pivotal roles of MHC–peptide binding and subsequent TCR–pMHC interactions. It then surveys the foundational public datasets that have fueled progress in this field, acknowledging their strengths and current limitations, particularly the challenges of data quality and quantity. Subsequently, the review details the diverse AI methodologies, with a particular focus on deep learning architectures, leveraged to predict these complex molecular interactions across MHC class I, MHC class II, and TCR–pMHC binding. While significant advancements are evident, marked by improved prediction accuracies, formidable challenges persist, including issues of data quality and scarcity, the need for enhanced algorithmic interpretability, and the pursuit of integrated “one-step” prediction models. Looking ahead, the future of AI-driven T-cell antigen identification is poised for continued growth, driven by three key interconnected pillars: benchmarking, computational methods, and wet experiment validation. This involves fostering standardized, high-throughput data generation, developing interpretable and multi-modal AI architectures (including MM, LLM, and GAI), and integrating functional immunogenicity data through “lab-in-the-loop” approaches. Ultimately, these innovations are paving the way for more rational mRNA vaccine design and personalized immunotherapies, promising to revolutionize the fight against infectious diseases and cancer.

**Key Points**

- This review provides a systematic categorization of artificial intelligence (AI)-driven computational methods for major histocompatibility complex (MHC)-I, MHC-II, and T-cell receptor (TCR)–peptide-MHC (pMHC) binding prediction, alongside a comprehensive survey of foundational data resources.
- A rigorous, standardized benchmarking of 18 state-of-the-art TCR–pMHC prediction models reveals a significant generalization gap when evaluated on out-of-distribution epitope variant datasets.
- The findings underscore a persistent challenge in structural modeling and generalization for novel epitope variants, highlighting the limitations of current sequence-based AI predictors.
- Future perspectives for immunoinformatics emphasize the integration of multi-omics data, enhanced structural modeling, and the development of generative models for *de novo* TCR design.

## Supplementary Material

Supplementary_materials_bbag123

## Data Availability

The resource, including code, and datasets, are freely available via GitHub at https://github.com/Sungden/AI-for-T-Cell-Antigen-Identification-Survey.
