# Supplementary material for: AI-driven computational methods and benchmarking for T-cell antigen identification
Source: Brief Bioinform. 2026 Mar 17;27(2):bbag123. doi: 10.1093/bib/bbag123 (PMC12993716; doi:10.1093/bib/bbag123)
Supplement: Supplementary_materials_bbag123 [file supplementary_materials_bbag123.pdf]

Table S1: Complete hyperparameter configurations for all 18 re-implemented TCR-pMHC binding prediction models.

**Model**	**Layers**	**Hidden Dim**	**Heads**	**Kernel Sizes**	**Key Parameters**
DeepTCR[19]	3 Conv + 3 FC	[32,64,128]	–	[5,3,3]	size_of_net='medium'
ERGO-II[20]	2 Bi-GRU	128	–	–	embed_dim=64
NetTCR-2.0[10]	Multi-kernel Conv	16	–	[1,3,5,7,9]	num_filters=16
ImRex[11]	3 Conv2D blocks	64	–	–	feat_mode='atchley'
TEIM[14]	3 Inter layers	128	–	–	inter_type='cat'
MixTCRpred [2]	6 Transformer	128	8	–	embedding_dim=128
pMTnet[9]	2 Conv + LSTM	30,16	–	[5,4]	tcr_embed_dim=30
UniPMT[31]	Residual MLPs	128	–	–	emb_size=64
PanPep[4]	1 Self-Attn + CNN	5	–	–	d_model=5
PISTE[3]	2 Enc + 2 Dec	64	4	–	d_model=64
TPepRet[25]	4 Transformer	128	8	–	d_model=128
TCRBagger[5]	1 Bi-LSTM	128	–	–	embed_dim=64
TEINet[7]	1 LSTM	64	–	–	cat_size=64
UnifyImmun[30]	Self-Attention	48	8	–	embed_dim=48
TITAN[26]	2 Bi-LSTM	128	4	–	hidden_dim=128
DLpTCR[29]	Ensemble	varies	–	varies	3 base models
PRIME 2.0[6]	5-layer MLP	128	–	–	token_emb=128
DeepAntigen[15]	5 GCN layers	128	4	–	hidden_dim=128

Table S2: Details of self-curated composite datasets referenced in Table 2.

**Model**	**Primary Task**	**Key Public Data Sources Integrated**
MixMHCpred2.2[6]	MHC-I ligand prediction	Immunopeptidomics MS data: Abelin et al. (2017) [1], Sarkizova et al. (2020) [17]; Binding affinity data from IEDB [24].
ImmuneApp[28]	MHC-I ligand prediction	Quantitative binding data from IEDB [24]; Immunopeptidomics data (e.g., Sarkizova et al. (2020) [17]); Functional epitope data from tumor neoantigen studies (e.g., TESLA consortium [27]).
MixMHCpred3.0 [22]	MHC-I ligand prediction	Expanded and updated immunopeptidomics MS datasets building upon MixMHCpred2.2 sources (e.g., [17, 1]) and additional recent studies.
MixMHC2pred2.0 [16]	MHC-II ligand prediction	MHC-II binding data from IEDB [24]; HLA-II immunopeptidomics data obtained by the authors via mass spectrometry.
NetMHCIIpan-4.2 [13]	MHC-II ligand prediction	Quantitative binding data from IEDB [24]; MHC-II eluted ligand MS data from studies like Stadinski et al.[21]
NetMHCIIpan-4.3 [12]	MHC-II ligand prediction	Builds upon NetMHCIIpan-4.2 sources, incorporating additional HLA-DQ/DP eluted ligand MS data.
PRIME2.0 [6]	TCR-pMHC recognition	Public TCR specificity databases: VDJdb [18], McPAS-TCR [23]; TCR-antigen pairing data from 10x Genomics platforms (e.g., MIRA dataset [8]).

References

[1] Jennifer G Abelin, Derin B Keskin, Siranush Sarkizova, Christina R Hartigan, Wandi Zhang, John Sidney, Jonathan Stevens, William Lane, Guang Lan Zhang, Thomas M Eisenhaure, et al. Mass spectrometry profiling of hla-associated peptidomes in mono-allelic cells enables more accurate epitope prediction. *Immunity*, 46(2):315–326, 2017.

[2] Giancarlo Croce, Sara Bobisse, Dana Léa Moreno, Julien Schmidt, Philippe Guillame, Alexandre Harari, and David Gfeller. Deep learning predictions of tcr-epitope interactions reveal epitope-specific chains in dual alpha T cells. *Nature Communications*, 15(1):3211, 2024.

[3] Ziyan Feng, Jingyang Chen, Youlong Hai, Xuelian Pang, Kun Zheng, Chenglong Xie, Xiujuan Zhang, Shengqing Li, Chengjuan Zhang, Kangdong Liu, et al. Sliding-attention transformer neural architecture for predicting t cell receptor–antigen–human leucocyte antigen binding. *Nature Machine Intelligence*, 6(10):1216–1230, 2024.

[4] Yicheng Gao, Yuli Gao, Yuxiao Fan, Chengyu Zhu, Zhiting Wei, Chi Zhou, Guohui Chuai, Qinchang Chen, He Zhang, and Qi Liu. Pan-peptide meta learning for t-cell receptor–antigen binding recognition. *Nature Machine Intelligence*, 5(3):236–249, 2023.

[5] Yuli Gao, Yicheng Gao, Siqi Wu, Danlu Li, Chi Zhou, Fangliangzi Meng, Kejing Dong, Xueying Zhao, Ping Li, Aibin Liang, et al. Weakly supervised peptide-tcr binding prediction facilitates neoantigen identification. *Cell Systems*, 16(10), 2025.

[6] David Gfeller, Julien Schmidt, Giancarlo Croce, Philippe Guillaume, Sara Bobisse, Raphael Genolet, Lise Queiroz, Julien Cesbron, Julien Racle, and Alexandre Harari. Improved predictions of antigen presentation and tcr recognition with mixmhcpred2. 2 and prime2. 0 reveal potent sars-cov-2 cd8+ t-cell epitopes. *Cell Systems*, 14(1):72–83, 2023.

[7] Yuepeng Jiang, Miaozhe Huo, and Shuai Cheng Li. Teinet: a deep learning framework for prediction of tcr–epitope binding specificity. *Briefings in bioinformatics*, 24(2):bbad086, 2023.

[8] Mark Klinger, François Pepin, Jordan Wilkins, Tom Asbury, Teresa Wittkop, Ji Zheng, Martin Moorhead, and Malek Faham. Multiplex identification of antigen-specific t cell receptors using a combination of immune assays and immune receptor sequencing. *PLOS ONE*, 10(10):e0141561, Oct 2015.

[9] Tianshi Lu, Ze Zhang, James Zhu, Yunguan Wang, Peixin Jiang, Xue Xiao, Chantale Bernatchez, John V Heymach, Don L Gibbons, Jun Wang, et al. Deep learning-based prediction of the t cell receptor–antigen binding specificity. *Nature machine intelligence*, 3(10):864–875, 2021.

[10] Alessandro Montemurro, Viktoria Schuster, Helle Rus Povlsen, Amalie Kai Bentzen, Vanessa Jurtz, William D Chronister, Austin Crinklaw, Sine R Hadrup, Ole Winther, Bjoern Peters, et al. Nettcr-2.0 enables accurate prediction of tcr-peptide binding by using paired tcrα and β sequence data. *Communications biology*, 4(1):1060, 2021.

[11] Pieter Moris, Joey De Pauw, Anna Postovskaya, Sofie Gielis, Nicolas De Neuter, Wout Bittremieux, Benson Ogunjimi, Kris Laukens, and Pieter Meysman. Current challenges for unseen-epitope tcr interaction prediction and a new perspective derived from image classification. *Briefings in bioinformatics*, 22(4):bbaa318, 2021.

[12] Jonas B Nilsson, Saghar Kaabinejadian, Hooman Yari, Michel GD Kester, Peter van Balen, William H Hildebrand, and Morten Nielsen. Accurate prediction of HLA class II antigen presentation across all loci using tailored data acquisition and refined machine learning. *Science Advances*, 9(47):eadj6367, 2023.

[13] Jonas Birkelund Nilsson, Saghar Kaabinejadian, Hooman Yari, Bjoern Peters, Carolina Barra, Loren Gragert, William Hildebrand, and Morten Nielsen. Machine learning reveals limited contribution of trans-only encoded variants to the HLA-DQ immunopeptidome. *Communications Biology*, 6(1):442, 2023.

[14] Xingang Peng, Yipin Lei, Peiyuan Feng, Lemei Jia, Jianzhu Ma, Dan Zhao, and Jianyang Zeng. Characterizing the interaction conformation between t-cell receptors and epitopes with deep learning. *Nature machine intelligence*, 5(4):395–407, 2023.

[15] Jinhao Que, Guangfu Xue, Tao Wang, Xiyun Jin, Zuxiang Wang, Yideng Cai, Wenyi Yang, Meng Luo, Qian Ding, Jinwei Zhang, et al. Identifying t cell antigen at the atomic level with graph convolutional network. *Nature Communications*, 16(1):5171, 2025.

[16] Julien Racle, Philippe Guillaume, Julien Schmidt, Justine Michaux, Amédé Larabi, Kelvin Lau, Marta AS Perez, Giancarlo Croce, Raphaël Genolet, George Coukos, et al. Machine learning predictions of MHC-II specificities reveal alternative binding mode of class II epitopes. *Immunity*, 56(6):1359–1375, 2023.

[17] Siranush Sarkizova, Susan Klaeger, Phuong M Le, Letitia W Li, Giacomo Oliveira, Hasmik Keshishian, Christina R Hartigan, Wandi Zhang, David A Braun, Keith L Ligon, et al. A large peptidome dataset improves hla class i epitope prediction across most of the human population. *Nature biotechnology*, 38(2):199–209, 2020.

[18] Mikhail Shugay, Dmitriy V. Bagaev, Ivan V. Zvyagin, Renske M. Vroomans, Jeremy C. Crawford, Garry Dolton, Ekaterina A. Komech, Anastasiya L. Sycheva, Anna E. Koneva, Evgeniy S. Egorov, Alexey V.

Eliseev, Erika Van Dyk, Pradyot Dash, Meriem Attaf, Cristina Rius, et al. Vdjdb: a curated database of t-cell receptor sequences with known antigen specificity. *Nucleic Acids Research*, 46(D1):D419–D427, Jan 2018.

[19] John-William Sidhom, H Benjamin Larman, Drew M Pardoll, and Alexander S Baras. Deeptcr is a deep learning framework for revealing sequence concepts within t-cell repertoires. *Nature communications*, 12(1):1605, 2021.

[20] Ido Springer, Nili Tickotsky, and Yoram Louzoun. Contribution of t cell receptor alpha and beta cdr3, mhc typing, v and j genes to peptide binding prediction. *Frontiers in immunology*, 12:664514, 2021.

[21] Brian D. Stadinski, Karthik Shekhar, Iria Gómez-Touriño, Jae Jung, Katsuyuki Sasaki, Andrew K. Sewell, Mark Peakman, Arup K. Chakraborty, and Eric S. Huseby. Hydrophobic cdr3 residues promote the development of self-reactive t cells. *Nature Immunology*, 17(8):946–955, Aug 2016.

[22] Daniel M Tadros, Julien Racle, and David Gfeller. Predicting MHC-I ligands across alleles and species: how far can we go? *Genome Medicine*, 17(1):25, 2025.

[23] Yuanjun Tong, Jie Wang, Tian Zheng, Xueying Zhang, Xian Xiao, Xiaoliang Zhu, Wei Lai, Lin Zeng, Mingwei Wang, and Kuan Xu. Mcpas-tcr: a manually curated catalogue of pathology-associated t cell receptor sequences. *Bioinformatics*, 34(17):2924–2929, Sep 2018.

[24] Randi Vita, Swapnil Mahajan, James A Overton, Sandeep Kumar Dhanda, Sheridan Martini, Jason R Cantrell, Daniel K Wheeler, Alessandro Sette, and Bjoern Peters. The immune epitope database (IEDB): 2018 update. *Nucleic Acids Research*, 47(D1):D339–D343, 2019.

[25] Meng Wang, Wei Fan, Tianrui Wu, and Min Li. Tpepret: a deep learning model for characterizing t-cell receptors–antigen binding patterns. *Bioinformatics*, 41(1):btaf022, 2025.

[26] Anna Weber, Jannis Born, and María Rodriguez Martínez. Titan: T-cell receptor specificity prediction with bimodal attention networks. *Bioinformatics*, 37(Supplement_1):i237–i244, 2021.

[27] Daniel K. Wells, Marijke M. van Buuren, Kristen K. Dang, Vanessa M. Hubbard-Lucey, Kathleen C. F. Sheehan, Katie M. Campbell, Andrew Lamb, Jeffrey P. Ward, John Sidney, Ana B. Blazquez, Andrew J. Rech, Mitchell R. Donovan, Philip D. Greenberg, Paul F. Robbins, Alessandro Sette, et al. Key parameters of tumor epitope immunogenicity revealed through a consortium approach improve neoantigen prediction. *Cell*, 183(3):818–834.e13, Oct 2020.

[28] Haodong Xu, Ruifeng Hu, Xianjun Dong, Lan Kuang, Wenchao Zhang, Chao Tu, Zhihong Li, and Zhongming Zhao. ImmuneApp for HLA-I epitope prediction and immunopeptidome analysis. *Nature Communications*, 15(1):8926, 2024.

[29] Zhaochun Xu, Meng Luo, Weizhong Lin, Guangfu Xue, Pingping Wang, Xiyun Jin, Chang Xu, Wenyang Zhou, Yideng Cai, Wenyi Yang, et al. Dlptcr: an ensemble deep learning framework for predicting immunogenic peptide recognized by t cell receptor. *Briefings in Bioinformatics*, 22(6), 2021.

[30] Chenpeng Yu, Xing Fang, Shiye Tian, and Hui Liu. A unified cross-attention model for predicting antigen binding specificity to both hla and tcr molecules. *Nature Machine Intelligence*, 7(2):278–292, 2025.

[31] Yunxiang Zhao, Jijun Yu, Yixin Su, You Shu, Enhao Ma, Jing Wang, Shuyang Jiang, Congwen Wei, Dongsheng Li, Zhen Huang, et al. A unified deep framework for peptide–major histocompatibility complex–t cell receptor binding prediction. *Nature Machine Intelligence*, pages 1–11, 2025.
